# Modeling and experimental approaches for elucidating multi-scale uterine smooth muscle electro- and mechano-physiology: A review

**DOI:** 10.3389/fphys.2022.1017649

**Published:** 2022-10-07

**Authors:** Amy S. Garrett, Shawn A. Means, Mathias W. Roesler, Kiara J. W. Miller, Leo K. Cheng, Alys R. Clark

**Affiliations:** Auckland Bioengineering Institute, University of Auckland, Auckland, New Zealand

**Keywords:** uterus, physiology, computational model, multiscale (MS) modeling, reproductive health, electrophysiology, uterus activation

## Abstract

The uterus provides protection and nourishment (via its blood supply) to a developing fetus, and contracts to deliver the baby at an appropriate time, thereby having a critical contribution to the life of every human. However, despite this vital role, it is an under-investigated organ, and gaps remain in our understanding of how contractions are initiated or coordinated. The uterus is a smooth muscle organ that undergoes variations in its contractile function in response to hormonal fluctuations, the extreme instance of this being during pregnancy and labor. Researchers typically use various approaches to studying this organ, such as experiments on uterine muscle cells, tissue samples, or the intact organ, or the employment of mathematical models to simulate the electrical, mechanical and ionic activity. The complexity exhibited in the coordinated contractions of the uterus remains a challenge to understand, requiring coordinated solutions from different research fields. This review investigates differences in the underlying physiology between human and common animal models utilized in experiments, and the experimental interventions and computational models used to assess uterine function. We look to a future of hybrid experimental interventions and modeling techniques that could be employed to improve the understanding of the mechanisms enabling the healthy function of the uterus.

## 1 Introduction

The human uterus undergoes dramatic functional changes: through monthly hormonal cycles throughout the lifetime of an individual, and in supporting a fetus in pregnancy. The uterus is a contractile smooth muscle organ, but the mechanisms that drive its contractile function are poorly understood. It can contract in the absence of neural or hormonal stimulation, but it undergoes intermittent periods of activation and relative quiescence throughout its lifespan. The most extreme example of this is in pregnancy, where a lack of contractions is required for most of gestation for the healthy growth of the fetus, and significant, coordinated contractions are required at parturition for successful labor and delivery (Rabotti and Mischi, 2015). However, significant patterns of contraction exist through the reproductive hormonal cycle (van Gestel et al., 2003), with the lowest amplitude and duration of contractions in the post-ovulatory phase and the highest at menstruation (in humans). There is variability in uterine activity, but typically by the second stage of labor contractions are expected to have a duration of 60 s, a frequency of 3-5 per 10 min, and amplitude of >50 mmHg (Bakker et al., 2007). A similar frequency might be expected during human menstruation, with durations of 30–60 s, and estimates of amplitude varying but ranging from 14 mmHg to as high as in early labor (van Gestel et al., 2003). Dysfunction of the uterine smooth muscle to initiate contractions in pregnancy can result in preterm labor and delivery, introducing health risk that can last a lifetime (Simmons et al., 2010; Crispi et al., 2018), and conditions such as endometriosis are hypothesized to be related to abnormal uterine contractions in the non-pregnant uterus (van Gestel et al., 2003).

**FIGURE 1:**
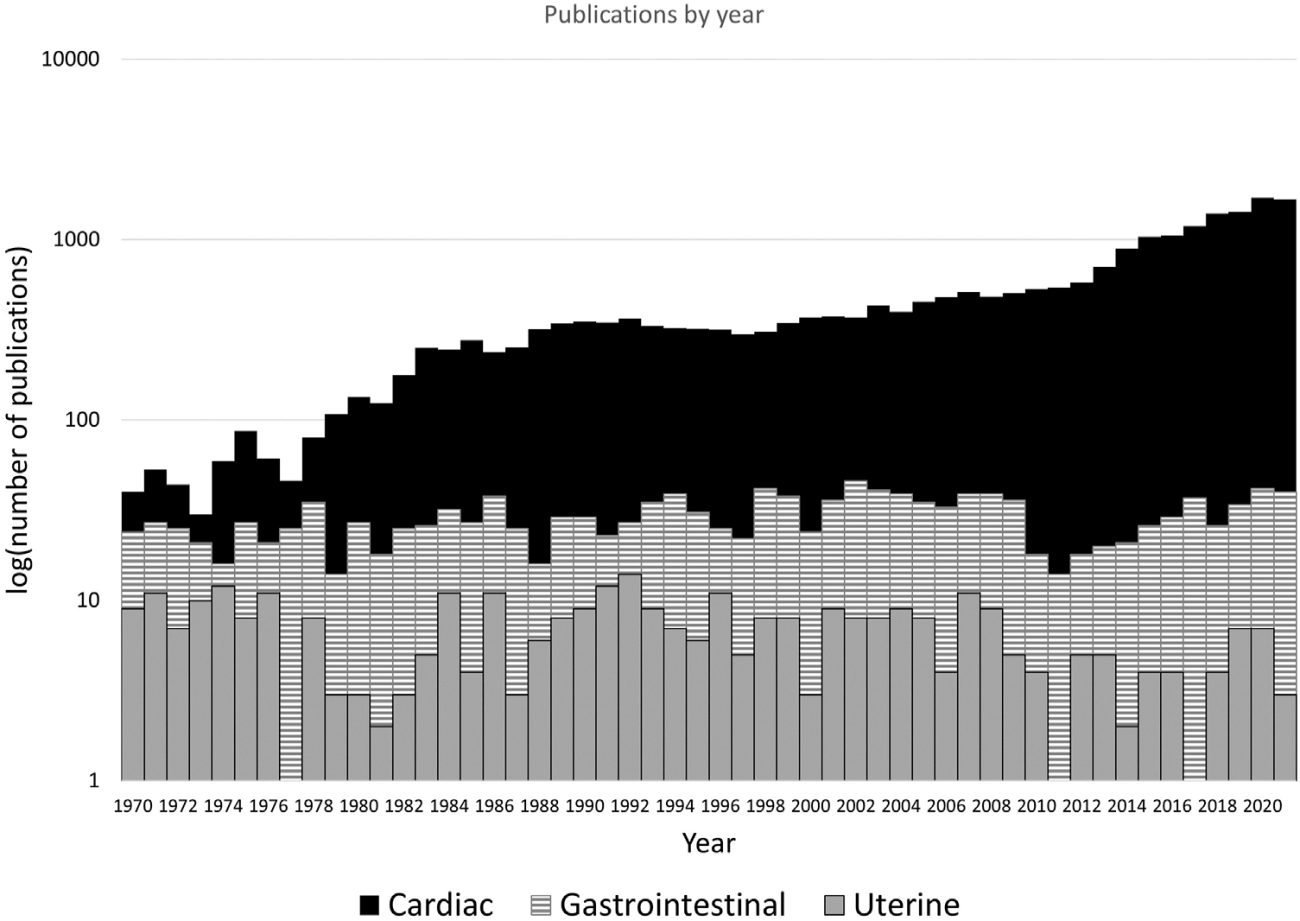
The results of a Pubmed (https://pubmed.ncbi.nlm.nih.gov/) search for “cardiac electrophysiology”, “gastrointestinal electrophysiology” and “uterine electrophysiology”, with variants of organ name (e.g., “uterus”) included. The logarithm of the number of articles per year between 1970 and 2021 are shown, as plotting number of publications rendered gastrointestinal and uterine data indistinguishable on the *y*-axis. Cardiac electrophysiology publications are increasing and exceeding 1,000 per year, gastrointestinal (smooth muscle organ) publications are steady at 20–40 per year. Publications in on uterine electrophysiology remain relatively stagnant, with <10 per year.

Coordinated contractions in a muscular organ are typically initiated and maintained by a region of pacemaker cells that modulate bioelectrical signals. For example, in the heart, a defined region of pacemaker cells in the atria initiates the spread of cardiac muscle contraction without failure in healthy individuals (Smith et al., 2015). Organs with smooth muscle, like the uterus, contract at a much lower frequency than the heart (in the order of minutes rather than seconds), but contractions can be sustained for longer periods. In the gastrointestinal system, electrical pacemaker function is coordinated by the interstitial cells of Cajal (ICCs), specialized neural interstitial cells which mediate coordinated contraction (Sanders, 1996). Similar cells in the uterus have been identified, named interstitial Cajal-like cells (ICLCs) or myometrial Cajal-like cells. Their scattered location throughout the uterine myometrium appears to indicate that, instead of a specific isolated pacemaker site, action potentials originate at various points throughout the organ (Lammers, 2013). The electrical activity required to enable the intense, coordinated contractions of labor is made possible by an increased proliferation of gap junctions, allowing a low resistance path of activation throughout the tissue (Miller et al., 1989). Despite the critical role of the uterus, particularly in pregnancy, and the evidence of unique features driving its electrophysiological function, its electrophysiology is poorly understood and lacks active research compared with cardiac or gastrointestinal electrophysiology (Figure 1).

To elucidate the electro-mechanical function of the non-pregnant and pregnant uterus, electrical mapping techniques can be utilized (Lammers et al., 2008; 2015; Lutton et al., 2018), as have previously been successfully applied to the heart (Durrer et al., 1970; Gepstein and Evans, 1998) and the gastrointestinal tract (Du et al., 2009; Lammers et al., 2009; O’Grady et al., 2010). Invasive measurements or experiments on samples of human tissue can be challenging from an ethical perspective, so frequently animal models aid in expanding physiological understanding and the development of measurement modalities and treatments. Bridging the gaps between animal experiments and human applications, mathematical modeling approaches provide insights into function that complement experimental studies. This review aims to provide an overview of key experimental and computational methods that have been used to investigate uterine smooth muscle electro-mechanical physiology in both pregnant and non-pregnant states. A particular focus is given to studies that aim to link the electrophysiology of the uterine smooth muscle cells to tissue or organ scale function.

## 2 Uterine physiology

Given the challenges involved with obtaining experimental data from human uteri, utilizing animal models for experimental investigation is critical. Understanding the structural and functional differences between species is therefore important to link experimental and modeling outcomes observed, as well as to aid translation of these data to the human context.

### 2.1 Physiology of the uterus between species

**FIGURE 2:**
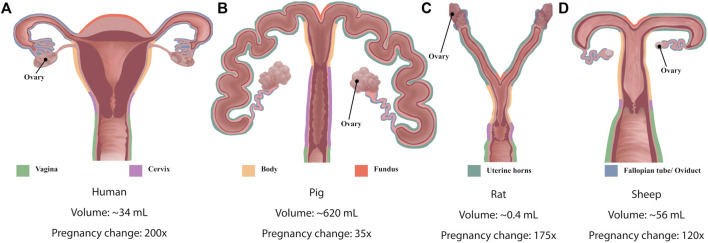
Schematic diagrams of the anatomy of **(A)** human uterus **(B)** pig, **(C)** rat and **(D)** sheep. Colored outlines denote different structures of the uteri. Diagrams shown not to scale. Indicative (approximate) uterine volumes provided, and the fold-change during pregnancy.

Significant species differences exist in the shape and function of the uterus (Figure 2 and Table 1). The human uterus is pyriform (pear-shaped); typically, a single fetus is carried for approximately 40 weeks (Malik et al., 2021). The human uterus (Figure 2A) comprises 4 regions: the fundus (upper edge), the body (middle section), leading down to the isthmus and cervix. In contrast, common experimental animals, such as pigs, sheep, and rodents, possess bicornuate uteri - with dual uterine horns. The two horns have multiple implantation sites, and these species have relatively short gestation periods (from 19 to 70 days, depending on the species) (Malik et al., 2021). Animal uteri typically also possess a smaller body and fundus than humans (Figures 2B–D). Although all mammals undergo hormonal cycles (estrous cycles) it is rare for a species to menstruate, as humans do (Profet, 1993). However, there are changes in the contractility of the uterus that have been observed with the estrous/menstrual cycle across species (Crane and Martin, 1991). The initiation of labor has been shown to relate to a progesterone withdrawal in many animal species, but not in humans (Mitchell and Taggart, 2009). The uterine smooth muscle cell also exhibits different biophysical profiles (e.g., expression of ion channels) between species, although a coordinated contraction is required for labor across animals (Mitchell and Taggart, 2009). In addition, differences in maturity at birth as well as litter numbers suggest that the mechanics of birth are species dependent, and studies of uterine function must consider these differences when interpreting data.

**TABLE 1:** Overview of species, physiology and experimental measures from the literature.

Animal	No. Offspring	Gestation length	Uterine shape	Measurement type	Sample type	References
Human	Typically 1	Typically 40 weeks	Pyriform	Contraction activity	Muscle strips	Fernandes and Crankshaw, 1995; Arrowsmith et al., 2018
				Contraction activity and pH	Muscle strip	Parratt et al. (1995)
				Contraction activity and Calcium	Muscle strip	Szal et al. (1994)
Pig	10–12	115–120 days	Bicornuate	Contraction activity	Muscle strips	Keye, 1923; Seckinger, 1923; Cao et al., 2002
Sheep	1–3	142–152 days	Bicornuate	Electrical activity	*In vivo* uterus	Parkington and Sigger, (1988)
				Electrical activity and intrauterine pressure	*In vivo* uterus	Verhoeff et al. (1985)
Guinea pig	2–4	59–72 days	Bicornuate	Electrical Activity	Intact uterus	Lammers et al. (2008)
					Muscle strips	Bozler, (1938b)
				Contraction activity	Motion Tracking	Lammers et al. (2015)
Rat	8–12	19–21 days	Bicornuate	Contraction activity	Muscle strips	Blair, 1923; Frank et al., 1925; (Durrant and Rosenfeld, 1931
				pH	Tissue samples	Wray, 1988b, 1990; Heaton et al., 1992
				Contraction activity and pH	Muscle strips	Taggart and Wray, 1993b, 1993a; Bullock et al., 1998
				Contraction activity and Calcium	Muscle strips	Taggart et al., 1996, 1997; Shmigol et al., 1998; Floyd et al., 2017
				Electrical Activity	*Ex vivo* uterus	Lammers et al., 1994; Lutton et al., 2018
					Muscle strips	Kuriyama and Suzuki, 1976; Lammers et al., 1995, 2015; Lammers, 1996; Lammers and Hamid, 1998
				Contraction activity and electrical activity	*Ex vivo* uterus with connection	Chkeir et al. (2013)
				Electrical activity and intrauterine pressure	*In vivo* uterus	de Paiva and Csapo, (1973)
				Electrical activity and tension	Muscle strips	Landa et al. (1959)
Mouse	7–12	19–21 days	Bicornuate	Contraction activity and Calcium	Muscle strips	Matthew et al. (2004)
				Contraction activity	Motion Tracking	Liang et al. (2019)
Rabbit	1–14	28–32 days	Bicornuate	Electrical activity	Muscle strips	Bozler, (1938b)
					*In vivo* uterus	Kao, (1959)
				Electrical activity and intrauterine pressure	*In vivo* uterus	Csapo et al., 1963; Csapo and Takeda, 1965

A commonality between species lies in the structural layers that comprise the uterine wall: the inner endometrium, the muscular myometrium, and the outer perimetrium. In the myometrium, myocytes bundle up to form two types of muscle layers: circumferential and longitudinal (Jain et al., 2000). The structure of these layers is clear in mammals with bicornuate uteri with longitudinal muscle fibers in the outer layer of the myometrium and circumferential fibers in the inner layer (Devedeux et al., 1993). However, the complex geometry of the human uterus presents a unique anisotropic fiber distribution throughout the myometrium and its various sublayers (Weiss et al., 2006), making the purely circumferential and longitudinal layers harder to distinguish (Wray and Prendergast, 2019; Malik et al., 2021). This difference in muscle fiber structure could be due to the differences in contraction mechanics required for the birthing of many young in animals compared with usually a single child for humans.

### 2.2 Uterine electrophysiology and smooth muscle function

Uterine smooth muscle cells (uSMCs) are specialized myocytes. There are two essential structures for contraction: the contractile mechanism and the sarcoplasmic reticulum (SR) (Jain et al., 2000). The contractile mechanism of myocytes is typically described by a sliding filament model comprised of three different myofilaments: 1) thick myosin filaments (15 nm in diameter), 2) thin actin filaments (8 nm in diameter), and 3) intermediate filaments (which form the structural network of the cell, 10 nm in diameter) (Verhoeff et al., 1985; Jain et al., 2000). The actin and intermediate filaments form a network connected via two structures: dense bands and dense bodies. The bands connect the network to the cell membrane, whereas the bodies connect actin to itself or intermediate filaments (Aguilar and Mitchell, 2010). Myofilaments and their associated dense bodies occupy 80–90% of the cell volume, with six times more actin than myosin in uterine SMCs (Aguilar and Mitchell, 2010).

During contraction, the myosin head binds to the actin filament, leading to cross-bridge cycling and the generation of tension as the myosin filament moves along the actin filament (Hafen and Burns, 2022). To initiate this process, a transient peak in calcium (Ca^2+^) closely precedes a peak in force production in a contraction. This Ca^2+^ binds to calmodulin, triggering the activation of myosin light-chain kinase (MLCK) and initiating the cross-bridge cycle (Wray, 2007). ATPase is important to catalyze the reaction (Malik et al., 2021; Wray and Arrowsmith, 2021), and the SR aids in the contraction process by releasing Ca^2+^ from its stores. This direct action of calcium binding to calmodulin in SMC is distinct from cardiac or skeletal striated muscle cells that require intermediates such as troponin for attachment of myosin heads. With smooth muscle, the actin-myosin duo can rest in a so-called ‘latch-state’ (Dillon et al., 1981), enabling maintenance of organ shape as well as contraction and movement with minimal energy expenditure.

**FIGURE 3:**
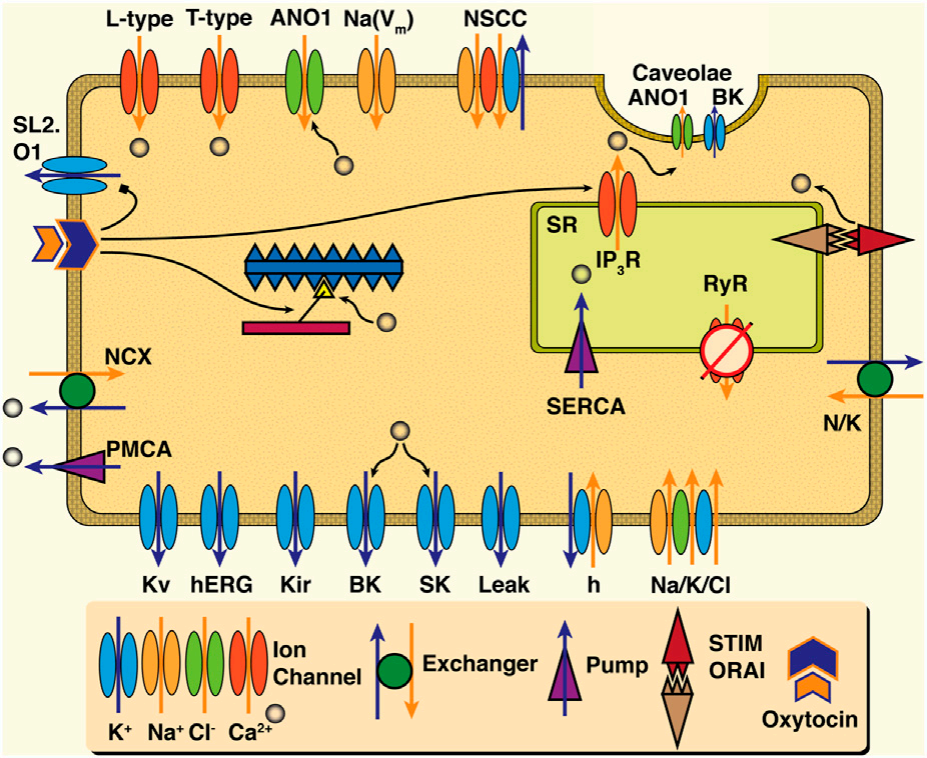
Schematic of cell dynamics relevant to uSMC. Ion channels ranging from the Ca^2+^ L-type to the wide array of K^+^ channels are present in the plasma membrane, and are colored by ion. Chloride channels such as the ANO1 appear not only on the plasma membrane but in the invaginations known as caveolae -- where Ca^2+^ activated K^+^ channels (the BK) reside in apposition with IP3R of the Sarcoplasmic Reticulum (SR). Ca^2+^ handling mechanisms for ejection to extracellular space (the NCX and PMCA) as well as uptake by the SR (SERCA) are present as well as the RyR with an apparently minor role if at all in uSMC. Action of hormonal oxytocin triggers variety of actions including release Ca^2+^ from SR by way of IP3R, inhibition of the otherwise hyperpolarizing K+ channel the SL2. O1, and sensitization of the contraction machinery to Ca^2+^ influence. Ultimately an increase in extra-cellular Ca^2+^ leads to activation of myosin light-chain kinase (MLCK) and initiates the cross bridge cycle which is shown in the center of the cell schematic.

Figure 3 illustrates the key cellular functions suggested to play a role in uSMC contraction. Tying Ca^2+^ and contraction to external signals are the ion fluxes transiting the plasma membrane. Local ionic currents generate action potentials that result in measurable electrophysiological signals. These signals can be observed at the tissue or organ scale as ‘waves’ of electrical propagation. The velocity of these waves varies from 10–45 mm s^−1^ depending on hormonal levels (Melton and Saldivar, 1964) and gestational day (Verhoeff et al., 1985). Ions flow in and out of the cell via various channels and exchangers detailed in recent reviews (Wray and Prendergast, 2019; Dunford et al., 2020; Wray and Arrowsmith, 2021). The Ca^2+^ required for myocyte, in general, controlled by ion channels and pumps (Aguilar and Mitchell, 2010). The voltage-gated ‘L-type’ channel is the primary mechanism for Ca^2+^ to enter the cell, which permits an influx of extracellular Ca^2+^ when the voltage gradient across the plasma membrane allows it (Shmigol et al., 1998; Wray et al., 2003). Other ions are also present in the cytosol or the intracellular space, including potassium (K^+^), sodium (Na^+^), and chloride (Cl^−^). K^+^ ions are primarily responsible for maintaining the negative resting membrane potential and repolarizing the cell (Wray and Prendergast, 2019), and Na^+^ ions slowly cause depolarization of the uSMCs during pregnancy (Amazu et al., 2020; Wray and Arrowsmith, 2021). The role of the Cl^−^ ions in uterine myocytes is unclear, but they may play a role in increasing excitability and contraction at term (Dunford et al., 2020).

The essential role of L-type channels in transporting Ca^2+^ across the plasma is well established via abolishment with nifedipine, ceasing contractile activity when applied (Shmigol et al., 1998; Wray et al., 2003). Meanwhile, the intracellular store of Ca^2+^ in the SR in the uterus likely plays a less dominant role in contraction (Shmigol et al., 2001; Wray and Prendergast, 2019), although it has a modulatory role (Noble et al., 2014). The SR shapes cytosolic Ca^2+^ signals with sarco-endoplasmic ATPase pumps (SERCA), removing Ca^2+^ along with the plasma-membrane ATPase pump (PMCA) and the sodium-calcium exchanger (NCX). Release of Ca^2+^ via the SR channels, the inositol-trisphosphate (IP3) receptor channel (IP3R) and ryanodine receptor (RyR) channel, initiate contraction, amongst other functions (Wray and Burdyga, 2010). The IP3R, triggered by oxytocin in clinical settings, release Ca^2+^ to induce stronger contractions (Arrowsmith and Wray, 2014) as well as inhibit a Na^+^-activated K^+^ channel (the SLo2.1) (Ferreira et al., 2019) and heighten response of contractile machinery to Ca^2+^ (Shmygol et al., 2006). Elevation of uSMC Ca^2+^ levels is also via store-operated calcium entry (the STIM-Orai mechanism; see (Putney, 2018) for a review) (Noble et al., 2014). Caveolae microdomains are populated with numerous ion channels such as the Ca^2+^-triggered chloride (ANO1), the large conductance potassium (the BK) (Babiychuk et al., 2004), and the store-operated calcium entry influx mechanism (Pani et al., 2008). They are also known to influence the essential L-type Ca^2+^ channels (Jacobo et al., 2009). Notably, excessive cholesterol overloads the lipid raft formations of caveolae, greatly diminishing the impact of oxytocin signaling strengths at these intracellular microdomains with pathological implications for the whole uterus (Smith et al., 2005). Therefore, despite the important role of L-type Ca^2+^, dynamics in uSMCs are modulated via a complex combination of pathways.

### 2.3 Uterine function in humans–Tools for assessment

Reliable measurement of intensity and time-course of uterine contraction during pregnancy and labor is key to diagnosing pre-term or dysfunctional labor (Xu et al., 2022). Outside of pregnancy, uterine monitoring can assess for abnormal uterine contractions that might indicate problems with fertility (Rabotti et al., 2015). The goal in both cases is organ-level uterine monitoring in clinical contexts. Uterine pressure can be directly measured using an intra-uterine pressure (IUP) catheter. IUP is the gold-standard measurement technique for uterine pressure. However, it is invasive and can only be achieved via rupturing the uterine membranes (Liston et al., 2007). This technique is reserved for labor, in necessary cases only, to reduce potential harmful side effects to both mother and fetus (Rabotti et al., 2008). Therefore, alternative, non-invasive tools are desirable.

The most common non-invasive clinical tool to monitor uterine activity is the tocodynamometer, a strain gauge attached to the abdominal surface that detects the motion of the abdominal muscles. This technology is useful in the final stages of pregnancy and during labor when the uterine wall is near the external abdomen. However, tocodynamometer measurements poorly correlate with underlying myometrial contractions and cannot quantify contraction intensity (Haran et al., 2012). The method relies on the contraction of the abdominal muscles as an indicator of global uterine pressure (Hadar et al., 2015).

Electrohysterography (EHG) is primarily a research focused non-invasive tool based on techniques developed for the heart (electrocardiography). It utilizes externally placed arrays of sensors originally developed to record maternal and fetal ECG signals to detect waves of electrical activity in the contracting uterus. The challenge lies in the signal processing techniques required to analyze the type of contractions and their propagation patterns, allowing for real-time analysis of uterine electrical activity (Rabotti et al., 2008; Rooijakkers et al., 2020). The overlap in frequency range between fetal heart rate, maternal heart rate and bursts of contraction activity can compound this issue. While EHG is readily applicable during pregnancy, in non-pregnant individuals, these electrical measurements are more difficult as the uterus sits deeper in the abdomen. The already small electrical signals become difficult to isolate, given the location and interference of other organs. However, EHG is favorable compared to external tocodynamometry (Thijssen et al., 2020) as it has consistently been shown to be more reliable, sensitive, and interpretable (Hayes-Gill et al., 2012; Vlemminx et al., 2017; Moni et al., 2021). Clinical review of uterine measurement techniques in labor (Cohen, 2017) and the application of EHG measurement for prediction of pre-term labor (Garcia-Casado et al., 2018) indicate that EHG shows promise for more routine use to assess uterine electrophysiology in the future.

Several commercially available EHG devices are currently on the market, including wireless systems (Table 2). Most wireless EHG measurement systems claim to be robust to maternal movements; however, the measurement context remains a clinical setting during labor. It is unclear whether these measurement systems would prevail in an external environment (e.g., at home) or work for a broader range of body types under non-clinical conditions. The advancement of measurement reliability could provide a useful at home tool for assessment of pregnancy and labor, and be used to inform clinical decisions.

**TABLE 2:** Commercially available EHG devices currently on the market, along with their ability to provide fetal heart rate (fHR), maternal heart rate (mHR).

Name	Website	fHR	mHR	EHG	Wireless	References
Bloomlife	https://bloomlife.com/	✗	✓	✓	✓	Altini et al. (2018)
PreTel	https://pretelhealth.com/	✓	✓	✓		
MindChild	https://www.mindchild.com/	✓	✗	✓	✗	
Novii	https://www.gehealthcare.com.au/products/maternal-infant-care/fetal-monitors/novii-wireless-patch-system	✓	✓	✓	✓	Hayes-Gill et al. (2012)
Avalon beltless fetal monitoring system	https://www.usa.philips.com/healthcare/product/HC866488/avalon-beltless-fetal-monitoring-solution	✓	✓	✓	✓	

## 3 Experimental measurements

**FIGURE 4:**
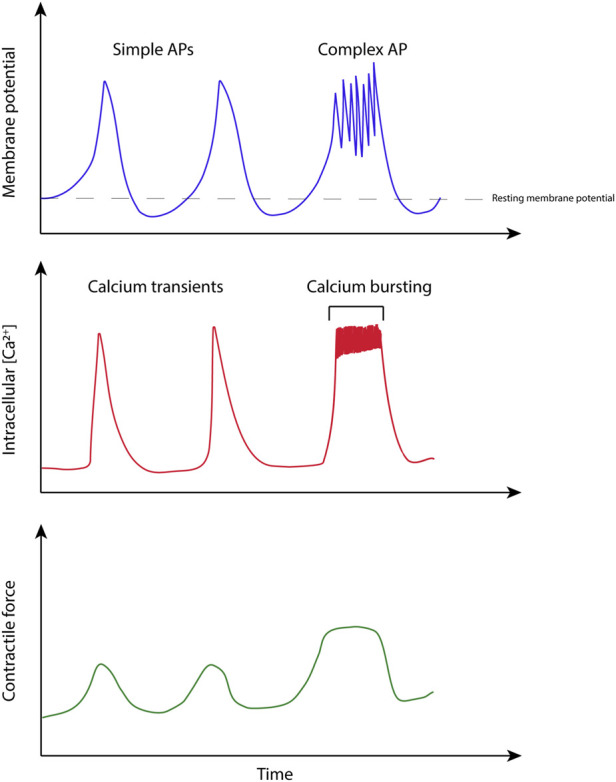
Illustration based on simultaneous measurements of electrical activity, calcium transients, and force in uterine smooth muscle. Adapted from experimental recordings from (Burdyga et al., 2007).

Development of technologies to monitor uterine function relies on an understanding of how the organ functions from an electro-mechanical perspective, across spatial scales, from sub-cellular function to the whole organ. The following sections outline the key experimental measurements which have been undertaken to date in uterine smooth muscle research, including active force, electrical activity arising from action potentials and ionic flux (specifically Ca^2+^). Figure 4 shows an example of the experimental measurement of these three key quantities.

### 3.1 Active force measurements in muscle

Like cardiac and skeletal muscle, the measurement of active force production in uSMC was first pioneered in the early 20th century (Blair, 1923; Keye, 1923; Seckinger, 1923). Force measurement techniques have been applied to study uSMC contractions, including the reaction of muscular mechanical activity to pharmacological interventions, to aid in the development of therapies to either halt contractions in early labor, or help advance difficult labor (Heaton et al., 1992; Matthew et al., 2004). This is viable for human tissue experiments and has been used to investigate changes in active mechanical contractions in both non-pregnant (Fernandes and Crankshaw, 1995) and pregnant myometrium (Arrowsmith et al., 2018). Contraction activity has also been assessed *ex vivo* using motion tracking algorithms. Spontaneous mechanical activity of *ex vivo* intact uteri can be recorded using high resolution cameras and the contractions quantified. In the isolated mouse uterus, the effects of muscle relaxant drugs for the development of treatments for painful menstrual cramps (dysmenorrhea), and in the isolated guinea pig uterus the location of a pacemaker regions have been investigated (Lammers et al., 2015; Liang et al., 2019). These studies focus on active force development in the muscle in response to stimuli, leaving passive mechanics (i.e., the baseline force or tension developed in the muscle in response to length changes) not included, although some work in passive length-tension has been carried out previously (Gordon and Siegman, 1971).

### 3.2 Intracellular pH measurements

Intracellular pH has long been known to influence smooth muscle contraction. Measurement in smooth muscle, including uterine myometrium, came into focus in the 1980s (Wray, 1988b). Nuclear Magnetic Resonance (P-NMR) spectroscopy measures intracellular pH by determining the compounds within the cell, mainly inorganic phosphate, whose resonant frequency is influenced by ph. Shifts in the frequency spectrum position of inorganic phosphate give an approximate measure of the intracellular pH, for example in response to changes in extracellular pH (Wray, 1988a). P-NMR allowed investigation of metabolic expenditure in pregnant and non-pregnant samples of uterine smooth muscle and the effect of metabolic inhibition (Wray, 1990). This technique opened the door for experiments that showed modulation of intracellular pH; using extracellular alkaline solutions results in increased contraction frequency of uterine myometrium (Heaton et al., 1992). Large volumes of tissue are required for accurate P-NMR measurements, and time resolution of measurement is poor, preventing simultaneous force measurements.

Fluorescent indicator techniques, for example, carboxy-SNARF, allowed simultaneous measurement of pH and force (Taggart and Wray, 1993b). When carboxy-SNARF is loaded into myometrial tissue strips and excited, intracellular pH is indicated by the ratio of two emission signals. Increased contraction force was observed with an increase in pH (alkalinization), and a decrease in force with a decrease in pH (acidification). This approach was used to investigate the small transient acidifications which accompany spontaneous contraction in uterine tissue (Taggart and Wray, 1993a), the buffering of pH between the extra- and intracellular environment (Bullock et al., 1998), and the implication of acidification during labor in human tissue (Parratt et al., 1995). The latter study found an increase in pH with gestation age, inferring that the alkalinization of tissue during pregnancy contributes to the increase in frequency and magnitude of contractions required at term.

### 3.3 Calcium measurements

pH modulation of force is closely tied to Ca^2+^, and Ca^2+^ measurement sheds light on the mechanism for uSMC contraction. Bioluminescence can be used to assess intracellular Ca^2+^ levels in muscle samples. A fluorescent molecule called aequorin (Jiang and Morgan, 1987), allowed the first simultaneous force and calcium measurement in uterine smooth muscle in intact (non-permeabilized) pregnant human uterine myometrium extracted during routine caesarean deliveries (Szal et al., 1994). This study showed that force production is dependent on calcium concentration, but also observed little change in the force-calcium relation in response to the use of agonist solutions. Taggart and Wray used aequorin calcium fluorescence to investigate the relationship between force, intracellular calcium and intracellular pH (Taggart et al., 1996), and the effect of phosphorylation inhibition with cyanide on the Ca^2+^-force relation (Taggart et al., 1997).

Over time, use of aequorin diminished, replaced with compounds such as Fura-2 and Indo-1 (Blinks, 1990). Fura-2 is a ratiometric indicator, where the ratio of the response to excitation at two wavelengths is related to the concentration of calcium in the cytoplasm. In comparison, Indo-1 fluorescence uses only one excitation wavelength, and quantifies the ratio of emission signals at two wavelengths. Although Indo-1 has been utilized in both human and animal samples to investigate a range of mechanisms (Shmigol et al., 1998; Matthew et al., 2004; Floyd et al., 2017), much is still unknown about the handling of calcium in uterine smooth muscle, although it is possible that the ‘energetics of calcium, homeostasis and force output in smooth muscle are independently regulated’, as succinctly stated by Taggart et al. (Taggart et al., 1997).

Along with calcium, the ionic flux of sodium and potassium have consequences during contraction. The influence of these ions in uterine smooth muscle can be measured using intracellular electrophysiology techniques, such as voltage clamp (Anderson, 1969; Mironneau, 1973; Reinl et al., 2015), current clamp (Anderson et al., 1971; Reinl et al., 2018) and patch clamp (Young et al., 1991; Young and Herndon-Smith, 1991). However, these measurement techniques are not a major focus of this review.

### 3.4 Electrophysiology mapping

Uterine electrophysiology is measured with electrodes placed on the surface or inserted in the organ, which record potential differences in membrane voltage. One of the first studies to record electrical activity in uSMC placed dissected strips of myometrial tissue in a muscle chamber with two types of electrodes: stimulating and recording. They showed that weak electric stimuli cause the uterine strips to contract, and the strips were able to contract spontaneously (Bozler, 1938a; 1938b). In this latter case, contractions were followed by bursts of electrical impulses. However, spontaneous contractions only occurred when the samples were extracted during estrus, with tissues extracted during diestrus exhibiting weak or no contractions. Since then, many investigators have used electrodes to measure uterine electrical activity. The protocol varies depending on if the experiment is conducted *ex vivo* (Lammers et al., 1994; Ma et al., 2020) or *in vivo* (Kao, 1959; Csapo et al., 1963). For *ex vivo* experiments, typically one of the uterine horns from small laboratory animals is excised and either kept intact (Csapo and Takeda, 1965; Lammers et al., 2008), opened along the mesometrial or anti-mesometrial border (Lammers et al., 2015; Lutton et al., 2018), or dissected to extract smaller segments of the myometrium (Landa et al., 1959; Kuriyama and Suzuki, 1976). However, maintaining the uterine horn intact allows for an *ex vivo* experiment to be conducted with the uterine horn in its hormonal environment (Chkeir et al., 2013). *In vivo* experiments are typically performed on larger animals, such as rabbits or sheep, with electrodes sutured into the uterine wall (Kao, 1959; Buhimschi and Garfield, 1996; Buhimschi et al., 1998). Some *in vivo* experiments have been conducted for several days, allowing for long-term observations of electrical activity (Kao, 1959; Verhoeff et al., 1985). Although most experiments measure spontaneous activity, the myometrium can be excited with a stimulating electrode (Parkington and Sigger, 1988).

Electrode studies have mainly focused on animals during pregnancy and near parturition, when the uterus is the most electrically active. Simultaneous recordings of the electrical activity and intra-uterine pressure indicate that electrical myometrial bursts, uterine volume and pressure are linked. An increase in volume triggers an increase in pressure and propagation of electrical bursts (Csapo and Kuriyama, 1963). As term approaches, the frequency of electric events increases, and their duration decreases (Verhoeff et al., 1985). Electrical bursts travel faster with a 2-fold increase during labor (Parkington and Sigger, 1988). Increases in propagation velocities have been linked to an increase in gap junction (Miller et al., 1989) and the presence of estradiol (Melton and Saldivar, 1964). Garfield et al. identified the formation of gap junctions in micrographs of uSMC when placed in tissue baths. They hypothesized that these were able to form as the tissue was no longer in contact with inhibitor hormones that block the synthesis of gap junctions (Garfield et al., 1980; Garfield and Hertzberg, 1990).

The development of high-resolution multi-electrode arrays in the 1990s has enabled the study of spatial patterns in the electrical activity of the uterus (Lammers et al., 1995; Lammers and Hamid, 1998; Chkeir et al., 2013). Arrays of up to 240 electrodes with a 2 mm inter-electrode distance enabled tracking electrical bursts in strips of myometrial tissue or across an entire uterine horn. This has prompted the search for a pacemaker area, a location where the electrical bursts originate, in the pregnant uterus (Lammers et al., 2015; Lutton et al., 2018). More recently, *in vivo* studies have been performed on non-gravid rat uteri, reporting spontaneous low frequency slow-wave type activity coupled with higher-frequency spike-like activity (Garrett et al., 2022).

There are few studies investigating the influence of the estrus cycle (diestrus/estrus) or during early pregnancy. Recently, Ma et al. identified patterns of coordinated activity in estrus and diestrus mice (Ma et al., 2020). They used a microelectrode array of 64 electrodes with an inter-electrode distance of 200 µm. During estrus, the activity in the strips was found to be synchronous and characterized by short bursts of high frequency and low amplitude. Contrarily, during diestrus, the activity was asynchronous and dominated by long-duration, low-frequency, and high-amplitude bursts.

The size of the tissue sample also affects experimental outcomes. Landa et al. noticed a correlation between electric spikes and mechanical contractions on 2 mm wide strips but not on those as wide as the horn (Landa et al., 1959). Similarly, Lammers et al. observed changes in the generation of spontaneous electric activity depending on the size of the myometrial strip. Generation became more regular when isolating a small strip of tissue from the organ, with a stable pacemaking site appearing (Lammers et al., 1994). The existence of a defined pacemaking site in the uterus remains contentious, and continual developments in both invasive and non-invasive monitoring of this function is needed.

## 4 Mathematically modeling uterine function

Mathematical modeling provides a flexible tool to complement experimental approaches, and to test hypotheses regarding uterine function where experiments cannot. This section outlines mathematical modeling techniques which attempt to address the complexities of the uterus over a range of scales, from the individual cell to tissue organization and characterize its function for the purpose of clinical applications.

### 4.1 Cellular functions

#### 4.1.1 Ion currents

Mathematical models of uSMC are natural derivatives of prior work investigating other SMC (Parthimos et al., 1999; Yang et al., 2003). The models differ in their level of detail describing the intracellular signaling pathways shown in Figure 3. We catalogue key models for the uSMC here. (For easy reference, an overview of these model features is provided in Supplementary Table S1) Of note, experimental data calibrating these models were primarily derived from published results from rat studies, except for one model (Atia et al., 2016). A seminal cell-level model of uSMC is the Bursztyn et al. model, which focuses on the relationship between membrane depolarization, intracellular Ca^2+^ triggered contraction, and force generation (Bursztyn et al., 2007). A key simplification of this model is that it utilizes an assumed functional form for membrane voltage (V_m_) independent of ionic current. This V_m_ parameter imparts its effect only on the L-type voltage-activated Ca^2+^ channel (see Supplementary Table S1), consistent with observations that this channel primarly influences uSMC contraction (Shmigol et al., 2001; Wray and Prendergast, 2019). However, dynamic control of membrane voltage beyond the flux of Ca^2+^ is necessary for activation (i.e., action potentials) and restoration of the system to baseline.

**FIGURE 5:**
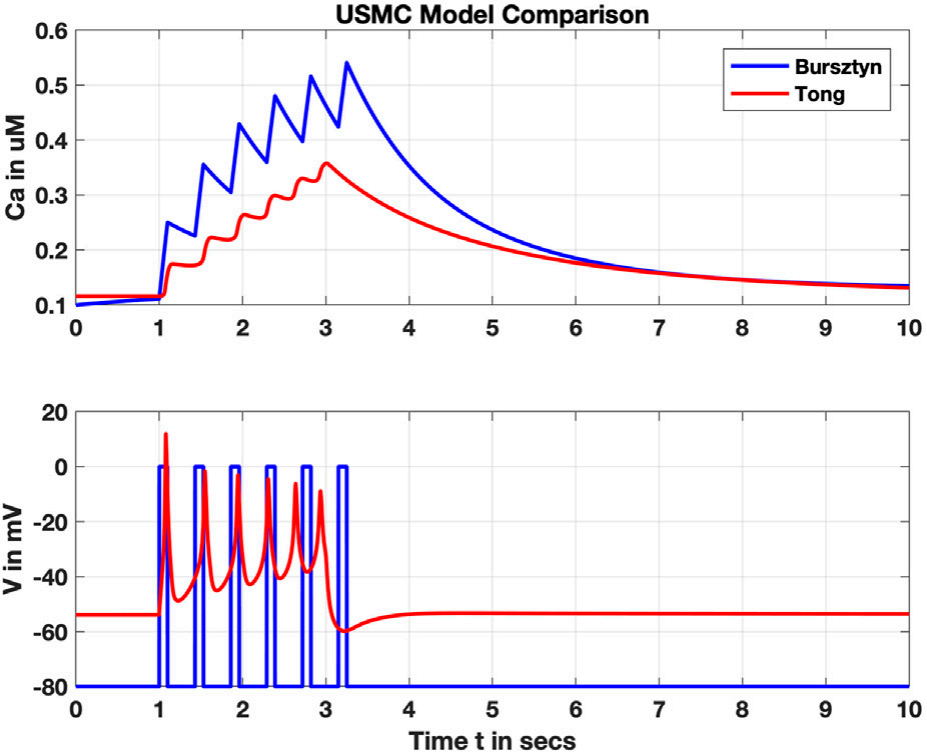
Comparison of the Bursztyn et al. and Tong et al. uSMC models. Shown are simulated intracellular calcium (Ca, upper panel) and membrane voltage (V, lower panel). V is an input parameter for the Bursztyn, whereas V is an output variable for the Tong responding to a steady stimulus current. Resulting Ca^2+^ concentrations are roughly comparable but with the simpler Bursztyn model rising higher likely due to lack of other currents such as the potassium suites included with the Tong model.

Subsequent models incorporated additional intracellular ions and their transporters, such as Rihana et al., adding K^+^ and Na^+^ (Rihana et al., 2009). Tong et al. later included 11 different ion channels in their model, adding chloride and non-specific cation channels with two subtypes of Ca^2+^-triggered K^+^ (Tong et al., 2011; 2014). The additional complexity of these channels led to distinct yet comparable results; see for instance a comparison of the Bursztyn and Tong models in Figure 5. In contrast to the Bursztyn model, the response of the Tong model is triggered by current injection, and therefore V_m_ is a model output. It takes approximately an order of magnitude longer to solve the Tong model (solved in OpenCOR, www.opencor.ws, using a CVODE solver), highlighting one tradeoff between physiological accuracy and complexity in uSMC modeling.

The most recent models of uSMC function have trended toward increased complexity moving towards a comprehensive model framework. Testrow et al. expanded the Tong model and investigated the influence of intracellular Ca^2+^ dynamics (Testrow et al., 2018). Meanwhile, approaching uSMC function from a transcriptomics perspective, Atia et al. identified mRNA sequences expressed in the uSMC and estimated parameters of the conductance repertoire (Atia et al., 2016). They proposed their complete collection of channels as ‘essential’ to uSMC function, with channel density estimated from mRNA data. However, they excluded some widely accepted channels in the uSMC, which appear in all other models. Potential targets for future investigation include the oxytocin-modulated potassium channel (SL2-01, see Figure 3), whose hyperpolarizing effect is reduced during oxytocin-mediated increased contraction (Ferreira et al., 2019).

#### 4.1.2 Ca^2+^ dynamics

Ca^2+^ is critical to uSMC contraction, with the L-type channels playing a major role (Shmigol et al., 2001; Wray and Prendergast, 2019). However, SR modulation via SERCA pumps and release of Ca^2+^ via the IP3R and RyR channels also influence contraction signals (see Figure 3) (Wray and Burdyga, 2010). While the SR plays a supportive role (Loftus et al., 2015; Wray and Prendergast, 2019), it is one with a potentially dramatic impact during pregnancy. Ca^2+^ dynamics are typically simplified representations in uSMC models, with only the classic 1977 (Wanner et al., 1977) and the recent Testrow et al. models including SR Ca^2+^ uptake and release (Testrow et al., 2018). It is important to consider similarities and differences between uSMC and other SMC. For example, the role of RyR in uSMC (if any) is likely less important than in other SMC (Matsuki et al., 2017), but it is nevertheless included in the Testrow model. IP3R, on the other hand, are present and clinically relevant during stimulation with oxytocin, but are strikingly excluded from any uSMC models to date. While the inclusion of some SR Ca^2+^ release mechanism is better than none, dynamical characteristics of these two SR channels are considerably different (Dupont et al., 2016).

In phasic smooth muscles SR IP3R channels are observed in tight proximity to caveolae, which are in turn populated with Ca^2+^-activated ANO1 and BK channels (Nixon et al., 1994). Spatial organization of L-type channels, which may also be located proximal to the SR (Navedo et al., 2005), add intrigue to the SR dynamic picture. The SR-plasma membrane Ca^2+^ microdomains and the remodeling of the uterine channel repertoires prior to and during parturition, which modulate or outright compete with the L-type channel dominance, appears territory ripe for mathematical exploration. However, most cell models focus primarily on the Ca^2+^-mediated connection between electrical propagation and contraction, an area considered next.

#### 4.1.3 Contraction within the cell

**FIGURE 6:**
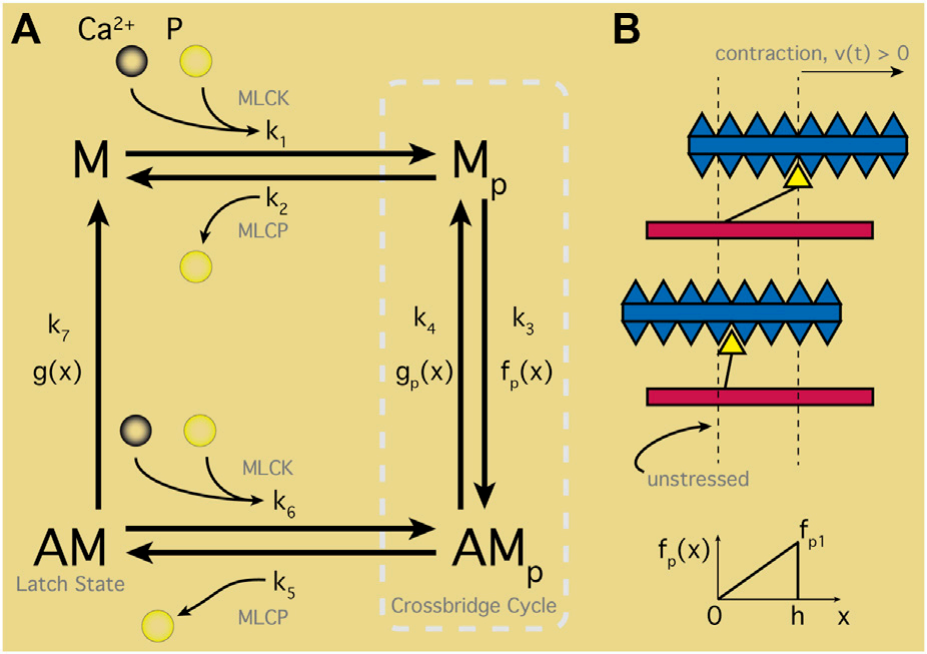
Smooth Muscle Contraction Schematic. **(A)** Four-states representing the myosin head (M) and actin filament/myosin complex (AM) in unphosphorylated states. Ca^2+^ binds with calmodulin-myosin complexes activating the myosin light-chain kinase (MLCK). MLCK combined with phosphate (*p*) transitions M to Mp, entering the crossbridge cycle with AMp. Transition between Mp and attached AMp states perform contractile work as determined by displacement, x. Rate of attachment in turn dependent on x with maximal and minimal displacements via f(x); detachment transitions similarly with g(x). Latch state—or dephosphorylated AM—classic feature of smooth muscle cells maintaining contracted state without energy input; note, M can only reach the latch state through the crossbridge cycle. **(B)** Depiction of contracted or unstressed latched states aligned with displacement range ( 0≤x≤h) and transition rate dependency on displacement illustrated for f(x) with maximum rate at full contraction. f(x) and g(x) designed to match Hai & Murphy parameters at the average point x = *h*/2.

Modeling of cellular contraction hinges on the action of Ca^2+^ interacting with the myosin-actin contractile machinery (Figure 6), typically based on the seminal model of Hai and Murphy (HM) for SMCs (Hai and Murphy, 1988). This model established a four-state scheme with the states representing the myosin head (M) and the actin myosin complex (AM) in both phosphorylated (AMp) and unphosphorylated states. Ordinary differential equations (ODEs) represent transitions between different states and reaction rates (the k_i_ in Figure 6) between each state are all constant with two exceptions: k_1_ and k_6._ Both are modulated by free Ca^2+^ driving phosphorylation by way of the MLCK (see Section 2.2). Instead of explicitly modeling this Ca^2+^ influence, HM used a time-dependent step function for k_1_ and k_6_ to mimic Ca^2+^ transients, with values fitted to phosphorylation data. Computing the force generated by the cross-bridge cycle entails displacement of the filaments. However, with the HM formulation there is effectively no displacement measured. Instead, stress (S) is defined as the total concentration of AM and AMp complexes. In uSMC modeling, Bursztyn et al. (Bursztyn et al., 2007) modified the HM model with fits of parameters to uterine data (Word et al., 1994). An explicit dependency of k_1_ on Ca^2+^ — a sigmoidal Hill function—was utilized instead of the time-dependent step function in the original HM model. This inspired incorporating the Ca^2+^ dynamics that Bursztyn merged into their model as described in Section 4.1.1.

Simulations of force generation computed with spatial displacement of myosin and actin filaments was introduced by (Fredberg et al., 1999; Mijailovich et al., 2000). These models hybridized the HM model and a Huxley sliding filaments model to produce the Huxley, Hai and Murphy (HHM) hybrid model (Huxely, 1957). HHM theory extended the HM ODE system to four partial differential equations (PDEs), where transition rates depended on displacements designed to match the average HM parameters (Figure 6B). Although more numerically complex to solve, the HHM model provides force calculations via the PDEs instead of the HM stress estimate, yet still limits Ca^2+^ impulses to a time-dependent step function from the original HM model (Hai and Murphy, 1988). Wang et al., later incorporated explicit Ca^2+^ dependencies into the HHM model (Wang et al., 2008) for arterial smooth muscle, including both forward phosphorylation transition of myosin and the reverse de-phosphorylation action. Additionally, the velocity of contraction, *v(t)*, appeared in the Wang model, exploiting the balance of force exertions by the SMC with surrounding elastic tissue in radially symmetric arteries.

These force calculation advances were later exploited for a uSMC model where Maggio et al. customized the HHM-Wang arterial SMC representation to uSMC, simplifying model dependency on Ca^2+^ in the control parameters for myosin light–chain phosphatase (Maggio et al., 2012), and using the same data (Word et al., 1994) fit by the Bursztyn model. This effort enabled prediction of force generation in the uSMC, investigating the sensitivity of the contraction shortening *v(t)* and its influence on isometry of contraction. The varying roles of uterine tissue during the process of facilitating menstruation, fertilization, or embryo transport could be critically dependent on this isometry. Maggio et al. defined a measure of isometry, the parameter gamma (*ɣ*), as the ratio of the average length of contractile units inside the cell to maximal attachment length (h) shown in Figure 6. A purely isometric contraction (*ɣ* = 0) generates the maximal possible contraction strength, according to Maggio et al. Alternatively, lower isometry (or increasing *ɣ*) weakens the peak contractile force due to greater disarray in the actin/myosin assemblies. Notably, Maggio showed that the slightly non-isometric *ɣ* in the HM overestimates the non-isometric strength, with implications for models using the HM scheme.

The later Testrow et al. model modified another arterial SMC contraction model from Yang et al. including passive elasticity of the cell, cross-bridge elasticity, and viscoelastic force (Yang et al., 2003). However, the Testrow et al. adaptation from the arterial suite of parameters to the uterine is quite limited; most values are simply carried over. Unfortunately, the literature on available parameters for the uSMC is not comprehensive, rather limiting utility of complex contraction models for uSMC despite their importance to characterizing uterine function. Ion channel parameters may be inferred analogous to the cardiac modeling effort of Ohara, et al., who isolated the distinct influences of membrane voltage and Ca^2+^ on inactivation of whole ventricular cells (O’Hara et al., 2011).

### 4.2 Modeling uterine tissue

Confocal microscopy images of Ca^2+^ transients in human myometrium demonstrated distinctive filamentary patterns in uterine tissue responding to stimuli (Bru-Mercier et al., 2012). The uSMC are thus organized into contractile fibers across the uterine tissue. A recent approach assembled the Testrow single-cell model, and their selected Tong and Yang submodels, into a fiber by constructing a linear array of individual uSMC (Goldsztejn and Nehorai, 2020). Their choice of the Yang submodel allows computation of the forces between individual cells and estimates of tension over the whole structure, for a constant total length. Electrical coupling is provided using the Tong solution for membrane voltage, V_m_, for each individual cell and the cable equation simulated 1D diffusion along the fiber. A resistance between the myocytes was applied, representing gap junctions in uterine tissue (Miyoshi et al., 1996). Goldzstejn et al. varied this intercellular resistance, inspecting its effect on conduction velocity over the idealized fiber, not unlike the observations of gap junction expression varying over the course of pregnancy and parturition (Miller et al., 1989; Balducci et al., 1993).

2D networks of myocytes assembled into relatively computationally inexpensive tissue models provide a different approach to understanding uterine tissue function. To achieve computational efficiency, typically full ion-current and membrane voltage simulations are neglected, and the classic Fitzhugh–Nagumo (FN) reduced model, with just two ODEs (an excitation and a recovery variable) is used (see Section 4.1.1). Benson et al. modified the FN two-variable ODE with a diffusion term for the excitation variable representing V_m_ (Benson et al., 2006). Since uterine tissue lacks a single specific pacemaker region (Lammers et al., 2015), Benson et al. randomly varied an input stimulus over a 2D spatially discretized square region with grid points treated as individual myocytes. Diffusive coupling between them was also randomly varied - representative of gap-junction variations. Heterogeneity was suggested key to overall behavior, driving transitions of the entire system between quiescence, bursting and synchrony. However, measured uSMC dynamic data did not inform their parameter set.

Sheldon et al. applied the FN excitable model for an idealized uterine tissue, but replaced diffusive coupling with a lattice network of point cells in a 25 × 25 grid (Sheldon et al., 2013). Positioned at the lattice center, a single pacemaker cell subjected all surrounding cells to its influence via random variations in coupling strength mimicking gap junctions. Sheldon et al. measured statistical distributions of capacitance and resting membrane potentials for murine uterine tissue at 18 days gestation and translated these measurements into distributions of coupling strengths and resting V_m_ over the lattice. Excitability for the entire system was largely dependent on the local correlations of coupling strength: lower correlations tended towards greater global excitation, reinforcing the heterogeneity results from the Benson model.

Xu et al. replaced the FN oscillator with the biophysical Tong et al. (2014) uSMC model and constructed a similar lattice-network tissue model (Xu et al., 2015). With a relatively large network of 50 × 50 cells, Xu et al. also varied coupling strength over the network, but further peppered random numbers of ‘ICC-like’ passive cells throughout the lattice. The ICC-like cell models excluded ion channel activity but coupled membrane voltage differences with surrounding uSMC cells in the lattice. Interestingly, no current was applied yet oscillations emerged: the uSMC cells experienced stimulation by way of the coupled passive cells given a high enough passive coupling strength (as previously seen by (Sheldon et al., 2013)). Progressively stronger coupling drove the system from quiescence to clusters of activity, followed by spatial waves across the lattice and eventually full synchronization. Qualitatively, the Xu model reproduced some behavior observed in cultures of uterine tissue (Lammers et al., 2008).

The utility of these tissue model explorations appears limited to feasibility studies–particularly given their disconnection with experimental data overall. Nevertheless, they permit consideration of distinct cells exhibiting varied intrinsic excitability (if any) and resting Vm along with distributions of coupling arrayed in idealized uterine tissue slices. Despite the lack of spatial scaling to actual uterine tissue, they provide insights into potential mechanisms generating the observed electrical patterns–but necessarily call for more realistic implementations.

Other groups have since expanded these concepts to larger networks of up to 10,000 cells in a multi-scaled scheme for simulating surface EHG signals (Rihana et al., 2009; Rabotti et al., 2010; Laforet et al., 2011). Overall, spatial coupling methods such as the gap-junction approach defined by Sheldon et al., the Koenigsberger geometric configuration (Koenigsberger et al., 2004) used by Laforet et al., or simply diffusion as in numerous tissue bidomain models, has built the groundwork for full-organ systems and the expansion to geometries, both simple and complex (Laforet et al., 2013)**.**

### 4.3 Whole organ modeling

Tissue level lattice models provide steps toward modeling clinical measurements such as EHG or IUP. However, the concepts explored in tissue level models (Section 4.2), need scaling up to the organ level to provide clinical interpretation. The earliest such models neglected the biophysical detail at the cell level with cellular automata schemes layered over ellipsoidal and more realistic uterine geometries (Andersen and Barclay, 1995; Young, 1997). By assuming a pacemaker region and varying its location Andersen and Barclay demonstrated geometric influence on tension in the system, with emergent tensile oscillations qualitatively similar to experimental patterns (Andersen and Barclay, 1995). By including calcium in addition to electrical propagation of action potentials along restricted paths (analogous to myocyte bundles from the fundus to the cervix) Young was able to generate simulations that fit measured IUP data (Young, 1997). The model restricted the APs to trigger calcium release in radially symmetric waves, confined to circular regions (presumed bounded by collagen fibers blocking progression). Ca^2+^ waves in turn switched cells from a resting to a contracted state, and the total number of contracted cells determined the force generated. The influence of structural organization on signaling was assessed by varying myocyte bundle size, Ca^2+^ wave speed and cellular contraction timespans (the only intracellular detail). Later, the same group considered mechano-transductive ion channels–a key and often overlooked element in the myometrium—which trigger spontaneous and phasic contractions upon stretching (Young, 2015). Rather than circular calcium waves they partitioned uterine tissue into electrically isolated regions (Ramon et al., 2005). Therefore, APs died out at distinct boundaries on the surface, assumed from non-conductive tissue or lack of gap junctions. The electrically isolated regions were coupled via mechano-transduction, transmitted through tension and pressure generated in the uterine volume. Variation of thresholds for regional excitations, as well as number of regions, suggested an optimal spatial partitioning for organ-wide response and synchronization with no explicit pacemaking region required, as had been assumed in earlier models (Andersen and Barclay, 1995).

**FIGURE 7:**
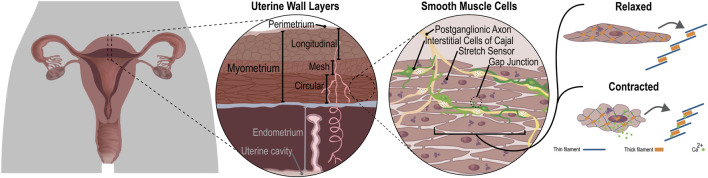
The multiple scales of uterine structure, ranging from the whole organ (left) to tissue layers, intra-tissue organization and individual cells (right). Characterizing uterine behavior entails teasing out dynamical connections between spatial and temporal scales such as myometrial responses to mechanical distensions shaped by tissue layers organized into contractile fibers. In turn, fibers comprised of individual cells coupled with neighbors via gap junctions or stretch activated sensors drive overall organ-wide responses from cell-level ion channel activity, themselves subject to hormonal influence either by endogenous evolution of concentrations up to parturition or artificially by clinical stimulation. Each scale individually presents mathematical modeling challenges, further amplified their integration into a whole, realistic and tractable representation of the entire organ.

**FIGURE 8:**
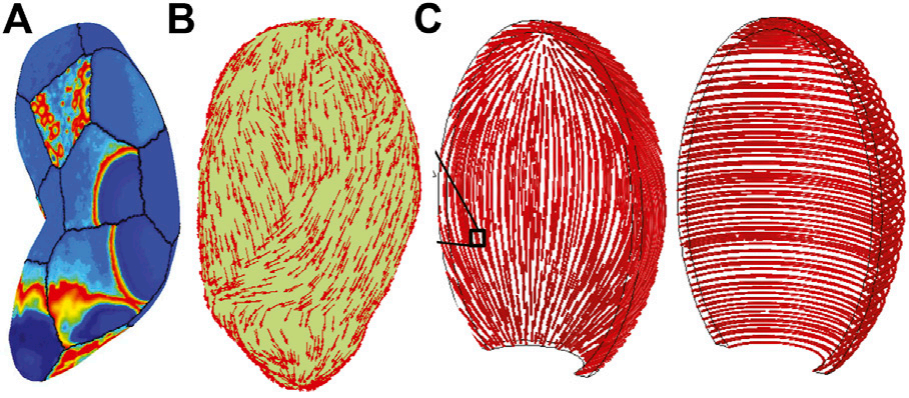
Three-dimensional organ simulations demonstrate tissue organizational dominant influence on overall behavior. **(A)** Yochum et al., simulation result showing electrical activity in spatially-distant regions due to mechano-transductive coupling well ahead of tissue-surface action potential wave-front (Yochum et al., 2018); note lack of contractile fibers over surface. **(B)** Organization of contractile fibers over three-dimensional organ simulated in Zhang et al., 2016, and layers of longitudinal and circumferential fiber layers (‘fasciculi’) in **(C)** Sharifmajid et al. (2016) inspired by observed patterns of fiber and collagen distributions of Weiss, et al., 2006. Reprinted from Yochum et al. (2018) with permission from Elsevier; Adapted from Zhang et al. (2016) under the Creative Commons License; Adapted from Sharifimajd et al. (2016) by permission from Springer Nature.

Notably, readily accessible EHG skin measurements are dependent on cellular mechanisms that determine transitions from a quiescent to contracting organ (Figure 7 illustrates connections between these scales). Influence of cellular scales on observable EHG may be simulated by solving the forward problem—projecting an electrical source through organ and tissue layers to the skin surface (Rabotti et al., 2010). Alternatively, bidomain formulations enable capturing essential cellular influences on the organ-wide analogous to those used in cardiac (Colli Franzone et al., 2005) and gastrointestinal models (Cheng et al., 2007; Sathar et al., 2015). These cumulated in the Yochum et al. (Yochum et al., 2018) comprehensive model of uterine electrophysiology, derived from an anatomic finite element model from FEMONUM repository (http://femonum.telecom-paristech.fr/) (Bibin et al., 2010) that included cellular contractile forces, as well as tissue elasticity and viscosity. Diffusion of membrane voltage over the surface was halted at boundaries between sub-divided regions of uterine surface (following (Young, 2015), see Figure 8A). Simulated mechano-transductive ion currents established a feedback mechanism between surface deformation and intracellular action potentials, with the model predicting that rapid increases in IUP do not occur without stretch-activation. Electrically isolated regions erupted into activity purely due to distortions sensed via mechano-transduction well ahead of depolarization wavefronts–evoking observations by (Eswaran et al., 2002) (Figure 8A). Transmission of contractions through the whole organ could thus be enabled by intracellular stretch-activated channels independently of V_m_.

With the aim of computational tractability, organ scale models of the uterus use simplifications in both the cellular models, and in the anatomy. Simplification of cellular dynamics to the FN model (Aslanidi et al., 2011) eases computational load at the whole organ scale in a realistic geometry. Cochran and Gao (2015) used the FN integrated into a bidomain tissue model for diffusive APs, and mechanical stress based on work in arterial wall mechanics (Holzapfel et al., 2000). Sharifimajd et al. later incorporated a Ca^2+^ model responsive to FN action potentials (Sharifimajd et al., 2016), and employed a HM contraction scheme with Ca^2+^-dependency for cross-bridge cycling (see Section 4.1.3). Each of these studies reproduced clinical and/or experimental data, despite differences in cell model formulation. Elucidating the relationship between cell and organ function in the uterus demands data driven approaches across spatial scales (Aslanidi et al., 2011), and potentially a drive for robust methods to simplify comprehensive cell level models. Balancing computational tractability and physiological relevance at the organ scale is essential.

Geometries ranging from both anatomically-realistic (Figures 8A,B, e.g., (Zhang et al., 2016)) to idealized ellipsoidal geometries (Figure 8C, e.g., (Sharifimajd et al., 2016)) appear in the modeling literature, and tissue fiber orientations vary widely as well. Interestingly, the same data describing fiber organization (Weiss et al., 2006), inspired two quite different anatomic models. La Rosa et al. imposed a uniform orientation angle of π/4 by virtue of a spherically symmetric geometry, identically aligning all fibers (la Rosa et al., 2012), yet Zhang et al., randomly perturbed orientation angles on a realistic geometry from a normal distribution over [0, π/4]; see Figure 8B (Zhang et al., 2016). Both studies, citing Weiss, demonstrated the primary influence of activity depends not on organ shape, but fiber orientation (Weiss et al., 2006). Human uterine fiber architecture is extraordinarily complex and further subject to change during pregnancy presenting challenges for not only organ-scale modeling, but measuring their likely distributions *in vivo*.

Numerous questions remain unanswered and multi-scale models are uniquely situated to address them, but finding simple, elegant and computationally tractable representations that capture essential behavior is key. Characterizing the fiber network influence on overall uterine function in a full model presents a formidable challenge, particularly considering stretch-activated electrical activity over disconnected regions. No single modeling scheme connects these two critical components to date, not to mention complications with including intracellular Ca^2+^ signaling details along with hormonal influences and phase of pregnancy. While, for instance, FN models indeed mitigate computational expense, the multi-pronged effects of oxytocin on release of Ca^2+^ through IP3R, combined with sensitization of cross-bridge phosphorylation and modulation of K^+^ channels, cannot be captured in such simple models. Integrating suitably complex cell-level models into tissue-wide and organ-scaled representations can benefit from experience with other organs such as cardiac or gastro-intestinal, but the subtle and unique complexity of the uterine system calls for novel and creative modeling solutions.

## 5 Conclusion

Despite the functional importance of the uterus, studies of the uterine electrophysiology lag those of other muscular organs. Studies of the uterus already incorporate methods from multiple arenas, such as experimental physiology and applied computational modeling, providing multiple avenues to capture the superficially simple yet exquisitely complex contractile activity of the uterine organ. Both experimental and mathematical techniques play a key role but together become more powerful. The influence of factors from cell signaling, through the complex contractile fiber orientations in the human uterus, to the effect of changes in organ anatomy through the menstrual cycle and pregnancy can only be achieved by a multi-scale analysis that includes both experimental and theoretical approaches. Mathematical model predictions are only as good as their parameter values, and fortunately, animal models provide data specific to the uterus to inform simulations, whereas human data is rare. Working in tandem, both can grapple with solving the enigma of uterine transition to powerful contractions after sustained quiescence. Identifying key metrics for detecting and preventing pathological pregnancies, particularly using non-invasive technologies, awaits as a target for future research.

## Data Availability

The implementations of the Bursztyn et al. and Tong et al. USMC models used to generate Figure 5 are available on the Physiome Model Repository (see https://models.physiomeproject.org/e/742 for the Bursztyn et al. model, and https://models.physiomeproject.org/e/263/for the Tong et al. model).

## Abbreviations

AM, Actin-myosin ‘Latch-State’; EHG, Electrohysterography; HM, Hai-Murphy Model; HHM, Huxley-Hai-Murphy Model; FN, Fitzhugh–Nagumo Model; ICC, Interstitial Cells of Cajal; IUP, Intrauterine Pressure; M, Mp, AMp Cross-bridge States; MLCK, Myosin Light-Chain Kinase; MLCP, Myosin Light-Chain Phosphatase; RyR, Ryanodine Receptor; SERCA, Sarcoplasmic/Endoplasmic Reticulum Calcium ATPase; SMC, Smooth Muscle Cell; uSMC, Uterine Smooth Muscle Cell.

## References

[B1] Aguilar H. N. Mitchell B. F. (2010). Physiological pathways and molecular mechanisms regulating uterine contractility. Hum. Reprod. Update 16, 725–744. 10.1093/humupd/dmq016 20551073

[B2] Altini M. Rossetti E. Rooijakkers M. J. Penders J. (2018). “Towards non-invasive labour detection: A free-living evaluation,” in Annu. Int. Conf. IEEE Eng. Med. Biol. Soc., 2018-July, 2841–2844. 10.1109/EMBC.2018.8512964 30440993

[B3] Amazu C. Ferreira J. J. Santi C. M. England S. K. (2020). Sodium channels and transporters in the myometrium. Curr. Opin. Physiol. 13, 141–144. 10.1016/j.cophys.2019.11.011 PMC11259238 39036486

[B4] Andersen H. F. Barclay M. L. (1995). A computer model of uterine contractions based on discrete contractile elements. Obstet. Gynecol. 86, 108–111. 10.1016/0029-7844(95)00111-4 7784002

[B5] Anderson N. C. Ramon F. Snyder A. (1971). Studies on calcium and sodium in uterine smooth muscle excitation under current-clamp and voltage-clamp conditions. J. Gen. Physiol. 58, 322–339. 10.1085/jgp.58.3.322 5095682 PMC2226030

[B6] Anderson N. C. (1969). Voltage-clamp studies on uterine smooth muscle. J. Gen. Physiol. 54, 145–165. 10.1085/jgp.54.2.145 5796366 PMC2225926

[B7] Arrowsmith S. Keov P. Muttenthaler M. Gruber C. W. (2018). Contractility measurements of human uterine smooth muscle to aid drug development. J. Vis. Exp. 131, e56639. 10.3791/56639 PMC5841565 29443077

[B8] Arrowsmith S. Wray S. (2014). Oxytocin: Its mechanism of action and receptor signalling in the myometrium. J. Neuroendocrinol. 26, 356–369. 10.1111/jne.12154 24888645

[B9] Aslanidi O. Atia J. Benson A. P. van den Berg H. A. Blanks A. M. Choi C. (2011). Towards a computational reconstruction of the electrodynamics of premature and full term human labour. Prog. Biophys. Mol. Biol. 107, 183–192. 10.1016/j.pbiomolbio.2011.07.004 21777604

[B10] Atia J. McCloskey C. Shmygol A. S. Rand D. A. van den Berg H. A. Blanks A. M. (2016). Reconstruction of cell surface densities of ion pumps, exchangers, and channels from mRNA expression, conductance kinetics, whole-cell calcium, and current-clamp voltage recordings, with an application to human uterine smooth muscle cells. PLoS Comput. Biol. 12, e1004828. 10.1371/journal.pcbi.1004828 27105427 PMC4841602

[B11] Babiychuk E. B. Smith R. D. Burdyga T. Babiychuk V. S. Wray S. Draeger A. (2004). Membrane cholesterol regulates smooth muscle phasic contraction. J. Membr. Biol. 198, 95–101. 10.1007/s00232-004-0663-1 15138749

[B12] Bakker P. C. A. M. van Rijsiwijk S. van Geijn H. P. van Geijn H. P. (2007). Uterine activity monitoring during labor. J. Perinat. Med. 35, 468–477. 10.1515/JPM.2007.116 18052832

[B13] Balducci J. Risek B. Gilula N. B. Hand A. Egan J. F. X. Vintzileos A. M. (1993). Gap junction formation in human myometrium: A key to preterm labor? Am. J. Obstet. Gynecol. 168, 1609–1615. 10.1016/S0002-9378(11)90806-0 8388630

[B14] Benson A. P. Clayton R. H. Holden A. V. Kharche S. Tong W. C. (2006). Endogenous driving and synchronization in cardiac and uterine virtual tissues: Bifurcations and local coupling. Philos. Trans. A Math. Phys. Eng. Sci. 364, 1313–1327. 10.1098/rsta.2006.1772 16608710

[B15] Bibin L. Anquez J. De La Plata Alcalde J. P. Boubekeur T. Angelini E. D. Bloch I. (2010). Whole-body pregnant woman modeling by digital geometry processing with detailed uterofetal unit based on medical images. IEEE Trans. Biomed. Eng. 57, 2346–2358. 10.1109/TBME.2010.2053367 20570763

[B16] Blair E. (1923). The contraction rate of the excised rat uterus with reference to the oestrous cycle. Am. J. Physiology-Legacy Content 65, 223–228. 10.1152/ajplegacy.1923.65.2.223

[B17] Blinks J. R. (1990). Use of photoproteins as intracellular calcium indicators. Environ. Health Perspect. 84, 75–81. 10.1289/ehp.908475 2190821 PMC1567652

[B18] Bozler E. (1938a). Electric stimulation and conduction of excitation in smooth muscle. Am. J. Physiology-Legacy Content 122, 614–623. 10.1152/ajplegacy.1938.122.3.614

[B19] Bozler E. (1938b). The action potentials of visceral smooth muscle. Am. J. Physiology-Legacy Content 124, 502–510. 10.1152/ajplegacy.1938.124.2.502

[B20] Bru-Mercier G. Gullam J. E. Thornton S. Blanks A. M. Shmygol A. (2012). Characterization of the tissue-level Ca2+ signals in spontaneously contracting human myometrium. J. Cell. Mol. Med. 16, 2990–3000. 10.1111/j.1582-4934.2012.01626.x 22947266 PMC4393727

[B21] Buhimschi C. Boyle M. B. Saade G. R. Garfield R. E. (1998). Uterine activity during pregnancy and labor assessed by simultaneous recordings from the myometrium and abdominal surface in the rat. Am. J. Obstet. Gynecol. 178, 811–822. 10.1016/S0002-9378(98)70498-3 9579450

[B22] Buhimschi C. Garfield R. E. (1996). Uterine contractility as assessed by abdominal surface recording of electromyographic activity in rats during pregnancy. Am. J. Obstet. Gynecol. 174, 744–753. 10.1016/S0002-9378(96)70459-3 8623816

[B23] Bullock A. J. Duquette R. A. Buttell N. Wray S. (1998). Developmental changes in intracellular pH buffering power in smooth muscle. Pflugers Arch. 435, 575–577. 10.1007/s004240050555 9446707

[B24] Burdyga T. Wray S. Noble K. (2007). “ *In situ* calcium signaling: No calcium sparks detected in rat myometrium,” in Annals of the New York academy of sciences. 10.1196/annals.1389.002 17303831

[B25] Bursztyn L. Eytan O. Jaffa A. J. Elad D. (2007). Mathematical model of excitation-contraction in a uterine smooth muscle cell. Am. J. Physiol. Cell Physiol. 292, C1816–C1829. 10.1152/ajpcell.00478.2006 17267547

[B26] Cao J. Shayibuzhati M. Tajima T. Kitazawa T. Taneike T. (2002). *In vitro* pharmacological characterization of the prostanoid receptor population in the non-pregnant porcine myometrium. Eur. J. Pharmacol. 442, 115–123. 10.1016/S0014-2999(02)01489-9 12020689

[B27] Cheng L. K. Komuro R. Austin T. M. Buist M. L. Pullan A. J. (2007). Anatomically realistic multiscale models of normal and abnormal gastrointestinal electrical activity. World J. Gastroenterol. 13, 1378–1383. 10.3748/wjg.v13.i9.1378 17457969 PMC4146922

[B28] Chkeir A. Fleury M.-J. Karlsson B. Hassan M. Marque C. (2013). Patterns of electrical activity synchronization in the pregnant rat uterus. Biomed. (Taipei) 3, 140–144. 10.1016/j.biomed.2013.04.007

[B29] Cochran A. L. Gao Y. (2015). A model and simulation of uterine contractions. Math. Mech. Solids 20, 540–564. 10.1177/1081286513507940

[B30] Cohen W. R. (2017). Clinical assessment of uterine contractions. Int. J. Gynaecol. Obstet. 139, 137–142. 10.1002/ijgo.12270 28727889

[B31] Colli Franzone P. Pavarino L. F. Taccardi B. (2005). Simulating patterns of excitation, repolarization and action potential duration with cardiac Bidomain and Monodomain models. Math. Biosci. 197, 35–66. 10.1016/j.mbs.2005.04.003 16009380

[B32] Crane L. H. Martin L. (1991). *In vivo* myometrial activity in the rat during the oestro us cycle: Studies with a novel technique of video laparoscopy. Reprod. Fertil. Dev. 3, 185–199. 10.1071/RD9910185 1835109

[B33] Crispi F. Miranda J. Gratacós E. (2018). Long-term cardiovascular consequences of fetal growth restriction: Biology, clinical implications, and opportunities for prevention of adult disease. Am. J. Obstet. Gynecol. 218, S869–S879. 10.1016/j.ajog.2017.12.012 29422215

[B34] Csapo A. I. Takeda H. (1965). Effect of progesterone on the electric activity and intrauterine pressure of pregnant and parturient rabbits. Am. J. Obstet. Gynecol. 91, 221–231. 10.1016/0002-9378(65)90204-8 14258024

[B35] Csapo A. I. Taskeda H. Wood C. (1963). Volume and activity of the parturient rabbit uterus. Am. J. Obstet. Gynecol. 85, 813–818. 10.1016/s0002-9378(16)35541-7 14024101

[B36] Csapo I. A. Kuriyama H. A. (1963). Effects of ions and drugs on cell membrane activity and tension in the postpartum rat myometrium. J. Physiol. 165, 575–592. 10.1113/jphysiol.1963.sp007081 14024103 PMC1359327

[B37] de Paiva C. E. N. Csapo A. I. (1973). The effect of prostaglandin on the electric activity of the pregnant uterus. Prostaglandins 4, 177–188. 10.1016/0090-6980(73)90037-3 4729609

[B38] Devedeux D. Marque C. Mansour S. Germain G. Duchêne J. (1993). Uterine electromyography: A critical review. Am. J. Obstet. Gynecol. 169, 1636–1653. 10.1016/0002-9378(93)90456-S 8267082

[B39] Dillon P. F. Aksoy M. O. Driska S. P. Murphy R. A. (1981). Myosin phosphorylation and the cross-bridge cycle in arterial smooth muscle. Science 211, 495–497. 10.1126/science.6893872 6893872

[B40] Du P. O’Grady G. Egbuji J. U. Lammers W. J. Budgett D. Nielsen P. (2009). High-resolution mapping of *in vivo* gastrointestinal slow wave activity using flexible printed circuit board electrodes: Methodology and validation. Ann. Biomed. Eng. 37, 839–846. 10.1007/s10439-009-9654-9 19224368 PMC4090363

[B41] Dunford J. R. Blanks A. M. Gallos G. (2020). Calcium activated chloride channels and their role in the myometrium. Curr. Opin. Physiol. 13, 43–48. 10.1016/j.cophys.2019.09.010

[B42] Dupont G. Falcke M. Kirk V. Sneyd J. (2016). Models of calcium signalling. Interdiscip. Appl. Math. 43, 1–436.

[B43] Durrant E. P. Rosenfeld S. (1931). Activity of the isolated uterus and its relation to the oestrous cycle in the albino rat. Am. J. Physiology-Legacy Content 98, 153–155. 10.1152/ajplegacy.1931.98.1.153

[B44] Durrer D. van Dam R. T. Freud G. E. Janse M. J. Meijler F. L. Arzbaecher R. C. (1970). Total excitation of the isolated human heart. Circulation 41, 899–912. 10.1161/01.cir.41.6.899 5482907

[B45] Eswaran H. Preissl H. Wilson J. D. Murphy P. Robinson S. E. Lowery C. L. (2002). First magnetomyographic recordings of uterine activity with spatial-temporal information with a 151-channel sensor array. Am. J. Obstet. Gynecol. 187, 145–151. 10.1067/mob.2002.123031 12114902

[B46] Fernandes B. Crankshaw D. (1995). Functional characterization of the prostanoid DP receptor in human myometrium. Eur. J. Pharmacol. 283, 73–81. 10.1016/0014-2999(95)00288-V 7498323

[B47] Ferreira J. J. Butler A. Stewart R. Gonzalez‐Cota A. L. Lybaert P. Amazu C. (2019). Oxytocin can regulate myometrial smooth muscle excitability by inhibiting the Na ^+^ ‐activated K ^+^ channel, Slo2.1. J. Physiol. 597, 137–149. 10.1113/JP276806 30334255 PMC6312452

[B48] Floyd R. V. Mobasheri A. Wray S. (2017). Gestation changes sodium pump isoform expression, leading to changes in ouabain sensitivity, contractility, and intracellular calcium in rat uterus. Physiol. Rep. 5. 10.14814/phy2.13527 PMC5727280 29208689

[B49] Frank R. T. Bonham C. D. Gustavson R. G. (1925). A new method of assaying the potency of the female sex hormone based upon its effect on the spontaneous contraction of the uterus of the white rat. Am. J. Physiology-Legacy Content 74, 395–399. 10.1152/ajplegacy.1925.74.2.395

[B50] Fredberg J. J. Inouye D. S. Mijailovich S. M. Butler J. P. (1999). Perturbed equilibrium of myosin binding in airway smooth muscle and its implications in bronchospasm. Am. J. Respir. Crit. Care Med. 159, 959–967. 10.1164/ajrccm.159.3.9804060 10051279

[B51] Garcia-Casado J. Ye-Lin Y. Prats-Boluda G. Mas-Cabo J. Alberola-Rubio J. Perales A. (2018). Electrohysterography in the diagnosis of preterm birth: A review. Physiol. Meas. 39, 02TR01. 10.1088/1361-6579/aaad56 29406317

[B52] Garfield R. E. Hertzberg E. L. (1990). Cell-to-cell coupling in the myometrium: Emil Bozler’s prediction. Prog. Clin. Biol. Res. 327, 673–681. 2181481

[B53] Garfield R. E. Merrett D. Grover A. K. (1980). Gap junction formation and regulation in myometrium. Am. J. Physiol. 8, C217–C228. 10.1152/ajpcell.1980.239.5.c217 7435609

[B54] Garrett A. S. Roesler M. W. Athavale O. N. Du P. Clark A. R. Cheng L. K. (2022). “ *In vivo* multi-channel measurement of electrical activity of the non-pregnant rat uterus,” in 2022 44th Annual International Conference of the IEEE Engineering in Medicine & Biology Society (EMBC), 3682–3685. 10.1109/EMBC48229.2022.9871943 36085904

[B55] Gepstein L. Evans S. J. (1998). Electroanatomical mapping of the heart: Basic concepts and implications for the treatment of cardiac arrhythmias. Pacing Clin. Electrophysiol. 21, 1268–1278. 10.1111/j.1540-8159.1998.tb00187.x 9633070

[B56] Goldsztejn U. Nehorai A. (2020). A myofibre model for the study of uterine excitation-contraction dynamics. Sci. Rep. 10, 16221. 10.1038/s41598-020-72562-x 33004882 PMC7530703

[B57] Gordon A. R. Siegman M. J. (1971). Mechanical properties of smooth muscle. I. Length-tension and force-velocity relations. Am. J. Physiol. 221, 1243–1249. 10.1152/ajplegacy.1971.221.5.1243 5124267

[B58] Hadar E. Biron-Shental T. Gavish O. Raban O. Yogev Y. (2015). A comparison between electrical uterine monitor, tocodynamometer and intra uterine pressure catheter for uterine activity in labor. J. Matern. Fetal. Neonatal Med. 28, 1367–1374. 10.3109/14767058.2014.954539 25123517

[B59] Hafen B. B. Burns B. (2022). Physiology, smooth muscle. Treasure Island, FL: StatPearls Publishing. 30252381

[B60] Hai C. M. Murphy R. A. (1988). Cross-bridge phosphorylation and regulation of latch state in smooth muscle. Am. J. Physiol. 254, C99–C106. 10.1152/ajpcell.1988.254.1.c99 3337223

[B61] Haran G. Elbaz M. Fejgin M. D. Biron-Shental T. (2012). A comparison of surface acquired uterine electromyography and intrauterine pressure catheter to assess uterine activity. Am. J. Obstet. Gynecol. 206, 412.e1–e5. e5. 10.1016/j.ajog.2011.12.015 22284960

[B62] Hayes-Gill B. Hassan S. Mirza F. G. Ommani S. Himsworth J. Solomon M. (2012). Accuracy and reliability of uterine contraction identification using abdominal surface electrodes. Clin. Med Insights Womens Health. 5, S10444. 10.4137/cmwh.s10444

[B63] Heaton R. C. Taggart M. J. Wray S. (1992). The effects of intracellular and extracellular alkalinization on contractions of the isolated rat uterus. Pflugers Arch gers Archiv Eur. J. Physiology 422, 24–30. 10.1007/BF00381509 1437523

[B64] Holzapfel G. A. Gasser T. C. Ogden R. W. (2000). A new constitutive framework for arterial wall mechanics and a comparative study of material models. J. Elast. 61, 1–48. 10.1023/A:1010835316564

[B65] Huxely A. F. (1957). Muscle structure and theories of contraction. Prog. Biophys. Biophys. Chem. 7, 255–318. 10.1016/s0096-4174(18)30128-8 13485191

[B66] Jacobo S. M. P. Guerra M. L. Jarrard R. E. Przybyla J. A. Liu G. Watts V. J. (2009). The intracellular II-III loops of Cav1.2 and Cav1.3 uncouple L-type voltage-gated Ca2+ channels from glucagon-like peptide-1 potentiation of insulin secretion in INS-1 cells via displacement from lipid rafts. J. Pharmacol. Exp. Ther. 330, 283–293. 10.1124/jpet.109.150672 19351867 PMC2700170

[B67] Jain V. Saade G. R. Garfield R. E. (2000). Structure and function of the myometrium. Adv. Organ Biol. 8, 215–246. 10.1016/s1569-2590(00)08009-5

[B68] Jiang M. J. Morgan K. G. (1987). Intracellular calcium levels in phorbol ester-induced contractions of vascular muscle. Am. J. Physiol. 253, H1365–H1371. 10.1152/ajpheart.1987.253.6.h1365 3425738

[B69] Kao C. Y. (1959). Long-term observations of spontaneous electrical activity of the uterine smooth muscle. Am. J. Physiol. 196, 343–350. 10.1152/ajplegacy.1959.196.2.343 13627177

[B70] Keye J. D. (1923). Periodic variations in spontaneous contractions of uterine muscle, in relation to the oestrous cycle and early pregnancy. Bull. Johns Hopkins Hosp. 34, 60–63.

[B71] Koenigsberger M. Sauser R. Lamboley M. Bény J. L. Meister J. J. (2004). Ca2+ dynamics in a population of smooth muscle cells: Modeling the recruitment and synchronization. Biophys. J. 87, 92–104. 10.1529/biophysj.103.037853 15240448 PMC1304399

[B72] Kuriyama H. Suzuki H. (1976). Changes in electrical properties of rat myometrium during gestation and following hormonal treatments. J. Physiol. 260, 315–333. 10.1113/jphysiol.1976.sp011517 978524 PMC1309093

[B73] la Rosa P. S. Eswaran H. Preissl H. Nehorai A. (2012). Multiscale forward electromagnetic model of uterine contractions during pregnancy. BMC Med. Phys. 12, 4. 10.1186/1756-6649-12-4 23126570 PMC3605117

[B74] Laforet J. Rabotti C. Mischi M. Marque C. (2013). Improved multi-scale modeling of uterine electrical activity. IRBM 34, 38–42. 10.1016/j.irbm.2012.12.004

[B75] Laforet J. Rabotti C. Terrien J. Mischi M. Marque C. (2011). Toward a multiscale model of the uterine electrical activity. IEEE Trans. Biomed. Eng. 58, 3487–3490. 10.1109/TBME.2011.2167970 21968708

[B76] Lammers W. J. El-Kays A. Arafat K. El-Sharkawy T. Y. (1995). Wave mapping: Detection of co-existing multiple wavefronts in high-resolution electrical mapping. Med. Biol. Eng. Comput. 33, 476–481. 10.1007/BF02510533 7666697

[B77] Lammers W. J. E. P. Arafat K. El-Kays A. El-Sharkawy T. Y. (1994). Spatial and temporal variations in local spike propagation in the myometrium of the 17-day pregnant rat. Am. J. Physiol. 267, C1210–C1223. 10.1152/ajpcell.1994.267.5.c1210 7977684

[B78] Lammers W. J. E. P. (1996). Circulating excitations and re-entry in the pregnant uterus. Pflugers Arch. 433, 287–293. 10.1007/s004240050279 9064644

[B79] Lammers W. J. E. P. Hamid R. (1998). The initiation, continuation, and termination of spontaneous episodes of circus movements in the pregnant myometrium of the rat. Am. J. Obstet. Gynecol. 179, 1515–1526. 10.1016/S0002-9378(98)70018-3 9855590

[B80] Lammers W. J. E. P. Mirghani H. Stephen B. Dhanasekaran S. Wahab A. Al Sultan M. A. H. H. (2008). Patterns of electrical propagation in the intact pregnant Guinea pig uterus. Am. J. Physiol. Regul. Integr. Comp. Physiol. 294, R919–R928. 10.1152/ajpregu.00704.2007 18046017

[B81] Lammers W. J. E. P. Stephen B. Al-Sultan M. A. Subramanya S. B. Blanks A. M. (2015). The location of pacemakers in the uteri of pregnant Guinea pigs and rats. Am. J. Physiol. Regul. Integr. Comp. Physiol. 309, R1439–R1446. 10.1152/ajpregu.00187.2015 26377559

[B82] Lammers W. J. E. P. (2013). The electrical activities of the uterus during pregnancy. Reprod. Sci. 20, 182–189. 10.1177/1933719112446082 22649122

[B83] Lammers W. J. E. P. Ver Donck L. Stephen B. Smets D. Schuurkes J. A. J. (2009). Origin and propagation of the slow wave in the canine stomach: The outlines of a gastric conduction system. Am. J. Physiol. Gastrointest. Liver Physiol. 296, G1200–G1210. 10.1152/ajpgi.90581.2008 19359425

[B84] Landa J. F. West T. C. Thiersch J. B. (1959). Relationships between contraction and membrane electrical activity in the isolated uterus of the pregnant rat. Am. J. Physiol. 196, 905–909. 10.1152/ajplegacy.1959.196.4.905 13637242

[B85] Liang K. L. Bursova J. O. Lam F. Chen X. Obukhov A. G. (2019). *Ex vivo* method for assessing the mouse reproductive tract spontaneous motility and a MATLAB-based uterus motion tracking algorithm for data analysis. J. Vis. Exp. 151, e59848 10.3791/59848 31524876

[B86] Liston R. Sawchuck D. Young D. Brassard N. Campbell K. Davies G. (2007). Fetal health surveillance: Antepartum and intrapartum consensus guideline. J. Obstetrics Gynaecol. Can. 29, S3–S4. 10.1016/S1701-2163(16)32615-9 17845745

[B87] Loftus F. C. Richardson M. J. E. Shmygol A. (2015). Single-cell mechanics and calcium signalling in organotypic slices of human myometrium. J. Biomech. 48, 1620–1624. 10.1016/j.jbiomech.2015.01.046 25702249 PMC4503816

[B88] Lutton E. J. Lammers W. J. E. P. James S. van den Berg H. A. Blanks A. M. (2018). Identification of uterine pacemaker regions at the myometrial–placental interface in the rat. J. Physiol. 596, 2841–2852. 10.1113/JP275688 29704394 PMC6046083

[B89] Ma X. Zhao P. Wakle-Prabagaran M. Amazu C. Malik M. Wu W. (2020). Microelectrode array analysis of mouse uterine smooth muscle electrical activity. Biol. Reprod. 102, 935–942. 10.1093/biolre/ioz214 31768528 PMC7124962

[B90] Maggio C. D. Jennings S. R. Robichaux J. L. Stapor P. C. Hyman J. M. (2012). A modified hai-murphy model of uterine smooth muscle contraction. Bull. Math. Biol. 74, 143–158. 10.1007/s11538-011-9681-1 21882077

[B91] Malik M. Roh M. England S. K. (2021). Uterine contractions in rodent models and humans. Acta Physiol. 231, e13607. 10.1111/apha.13607 PMC8047897 33337577

[B92] Matsuki K. Takemoto M. Suzuki Y. Yamamura H. Ohya S. Takeshima H. (2017). Ryanodine receptor type 3 does not contribute to contractions in the mouse myometrium regardless of pregnancy. Pflugers Arch. 469, 313–326. 10.1007/s00424-016-1900-z 27866274

[B93] Matthew A. Kupittayanant S. Burdyga T. Wray S. (2004). Characterization of contractile activity and intracellular Ca2+ signalling in mouse myometrium. J. Soc. Gynecol. Investig. 11, 207–212. 10.1016/j.jsgi.2003.10.009 15120693

[B94] Melton C. E. Saldivar J. T. (1964). Impulse velocity and conduction pathways in rat myometrium. Am. J. Physiol. 207, 279–285. 10.1152/ajplegacy.1964.207.2.279 14205336

[B95] Mijailovich S. M. Butler J. P. Fredberg J. J. (2000). Perturbed equilibria of myosin binding in airway smooth muscle: Bond-length distributions, mechanics, and ATP metabolism. Biophys. J. 79, 2667–2681. 10.1016/S0006-3495(00)76505-2 11053139 PMC1301147

[B96] Miller S. M. Garfield R. E. Daniel E. E. (1989). Improved propagation in myometrium associated with gap junctions during parturition. Am. J. Physiol. 256, C130–C141. 10.1152/ajpcell.1989.256.1.c130 2912131

[B97] Mironneau J. (1973). Excitation‐contraction coupling in voltage clamped uterine smooth muscle. J. Physiol. 233, 127–141. 10.1113/jphysiol.1973.sp010301 4796671 PMC1350543

[B98] Mitchell B. F. Taggart M. J. (2009). Are animal models relevant to key aspects of human parturition? Am. J. Physiol. Regul. Integr. Comp. Physiol. 297, R525–R545. 10.1152/ajpregu.00153.2009 19515978

[B99] Miyoshi H. Boyle M. B. MacKay L. B. Garfield R. E. (1996). Voltage-clamp studies of gap junctions between uterine muscle cells during term and preterm labor. Biophys. J. 71, 1324–1334. 10.1016/S0006-3495(96)79332-3 8874006 PMC1233599

[B100] Moni S. S. Kirshenbaum R. Comfort L. Kuba K. Wolfe D. Xie X. (2021). Noninvasive monitoring of uterine electrical activity among patients with obesity: A new external monitoring device. Am. J. Obstet. Gynecol. MFM 3, 100375. 10.1016/j.ajogmf.2021.100375 33852969

[B101] Navedo M. F. Amberg G. C. Votaw V. S. Santana L. F. (2005). Constitutively active L-type Ca2+ channels. Proc. Natl. Acad. Sci. U. S. A. 102, 11112–11117. 10.1073/pnas.0500360102 16040810 PMC1180225

[B102] Nixon G. F. Mignery G. A. Somlyo A. V. (1994). Immunogold localization of inositol 1, 4, 5-trisphosphate receptors and characterization of ultrastructural features of the sarcoplasmic reticulum in phasic and tonic smooth muscle. J. Muscle Res. Cell Motil. 15, 682–700. 10.1007/BF00121075 7706424

[B103] Noble D. Borysova L. Wray S. Burdyga T. (2014). Store-operated Ca²⁺ entry and depolarization explain the anomalous behaviour of myometrial SR: Effects of SERCA inhibition on electrical activity, Ca²⁺ and force. Cell Calcium 56, 188–194. 10.1016/j.ceca.2014.07.003 25084623 PMC4169181

[B104] O’Grady G. Du P. Cheng L. K. Egbuji J. U. Lammers W. J. Windsor J. A. (2010). Origin and propagation of human gastric slow-wave activity defined by high-resolution mapping. Am. J. Physiol. Gastrointest. Liver Physiol. 299, G585–G592. 10.1152/ajpgi.00125.2010 20595620 PMC2950696

[B105] O’Hara T. Virág L. Varró A. Rudy Y. (2011). Simulation of the undiseased human cardiac ventricular action potential: Model formulation and experimental validation. PLoS Comput. Biol. 7, e1002061. 10.1371/journal.pcbi.1002061 21637795 PMC3102752

[B106] Pani B. Hwei L. O. Liu X. Rauser K. Ambudkar I. S. Singh B. B. (2008). Lipid rafts determine clustering of STIM1 in endoplasmic reticulum-plasma membrane junctions and regulation of store-operated Ca2+ entry (SOCE). J. Biol. Chem. 283, 17333–17340. 10.1074/jbc.M800107200 18430726 PMC2427359

[B107] Parkington H. C. Sigger J. N. (1988). Co-ordination of electrical activity in the myometrium of pregnant ewes. J. Reprod. Fertil. 82, 697–705. 10.1530/jrf.0.0820697 3361503

[B108] Parratt J. R. Taggart M. J. Wray S. (1995). Functional effects of intracellular pH alteration in the human uterus: Simultaneous measurements of pH and force. J. Reprod. Fertil. 105, 71–75. 10.1530/jrf.0.1050071 7490717

[B109] Parthimos D. Edwards D. H. Griffith T. M. (1999). Minimal model of arterial chaos generated by coupled intracellular and membrane Ca2+ oscillators. Am. J. Physiol. 277, H1119–H1144. 10.1152/ajpheart.1999.277.3.h1119 10484436

[B110] Profet M. (1993). Menstruation as a defense against pathogens transported by sperm. Q. Rev. Biol. 68, 335–386. 10.1086/418170 8210311

[B111] Putney J. W. (2018). Forms and functions of store-operated calcium entry mediators, STIM and Orai. Adv. Biol. Regul. 68, 88–96. 10.1016/j.jbior.2017.11.006 29217255 PMC5955777

[B112] Rabotti C. Mischi M. Beulen L. Oei G. Bergmans J. W. M. (2010). Modeling and identification of the electrohysterographic volume conductor by high-density electrodes. IEEE Trans. Biomed. Eng. 57, 519–527. 10.1109/TBME.2009.2035440 19884073

[B113] Rabotti C. Mischi M. (2015). Propagation of electrical activity in uterine muscle during pregnancy: A review. Acta Physiol. 213, 406–416. 10.1111/apha.12424 25393600

[B114] Rabotti C. Mischi M. Van Laar J. O. E. H. Oei G. S. Bergmans J. W. M. (2008). Estimation of internal uterine pressure by joint amplitude and frequency analysis of electrohysterographic signals. Physiol. Meas. 29, 829–841. 10.1088/0967-3334/29/7/011 18583724

[B115] Rabotti C. Sammali F. Kuijsters N. Schoot B. Kortenhorst M. Mischi M. (2015). “Analysis of uterine activity in nonpregnant women by electrohysterography: A feasibility study,” in Proceedings of the Annual International Conference of the IEEE Engineering in Medicine and Biology Society, EMBS, 2015-Novem, 5916–5919. 10.1109/EMBC.2015.7319738 26737638

[B116] Ramon C. Preissl H. Murphy P. Wilson J. D. Lowery C. Eswaran H. (2005). Synchronization analysis of the uterine magnetic activity during contractions. Biomed. Eng. Online 4, 55. 10.1186/1475-925X-4-55 16197557 PMC1266387

[B117] Reinl E. L. Cabeza R. Gregory I. A. Cahill A. G. England S. K. (2015). Sodium leak channel, non-selective contributes to the leak current inhuman myometrial smooth muscle cells from pregnant women. Mol. Hum. Reprod. 21, 816–824. 10.1093/molehr/gav038 26134120 PMC4586347

[B118] Reinl E. L. Zhao P. Wu W. Ma X. Amazu C. Bok R. (2018). Na + -leak channel, non-selective (NALCN) regulates myometrial excitability and facilitates successful parturition. Cell. Physiol. biochem. 48, 503–515. 10.1159/000491805 30021195 PMC9639199

[B119] Rihana S. Terrien J. Germain G. Marque C. (2009). Mathematical modeling of electrical activity of uterine muscle cells. Med. Biol. Eng. Comput. 47, 665–675. 10.1007/s11517-009-0433-4 19301052

[B120] Rooijakkers M. J. Rabotti C. Oei S. G. Mischi M. (2020). Critical analysis of electrohysterographic methods for continuous monitoring of intrauterine pressure. Math. Biosci. Eng. 17, 3019–3039. 10.3934/mbe.2020171 32987514

[B121] Sanders K. M. (1996). A case for interstitial cells of Cajal as pacemakers and mediators of neurotransmission in the gastrointestinal tract. Gastroenterology 111, 492–515. 10.1053/gast.1996.v111.pm8690216 8690216

[B122] Sathar S. Trew M. L. O’Grady G. Cheng L. K. (2015). A multiscale tridomain model for simulating bioelectric gastric pacing. IEEE Trans. Biomed. Eng. 62, 2685–2692. 10.1109/TBME.2015.2444384 26080372 PMC4655104

[B123] Seckinger D. L. (1923). Spontaneous contractions of the Fallopian tube of the domestic pig with reference to the oestrous cycle. Bull. Johns Hopkins Hosp. 34.

[B124] Sharifimajd B. Thore C. J. Stålhand J. (2016). Simulating uterine contraction by using an electro-chemo-mechanical model. Biomech. Model. Mechanobiol. 15, 497–510. 10.1007/s10237-015-0703-z 26162461

[B125] Sheldon R. E. Baghdadi M. McCloskey C. Blanks A. M. Shmygol A. van den Berg H. A. (2013). Spatial heterogeneity enhances and modulates excitability in a mathematical model of the myometrium. J. R. Soc. Interface 10, 20130458. 10.1098/rsif.2013.0458 23843249 PMC3730699

[B126] Shmigol A. V. Eisner D. A. Wray S. (1998). Properties of voltage-activated [Ca2+](i) transients in single smooth muscle cells isolated from pregnant rat uterus. J. Physiol. 511, 803–811. 10.1111/j.1469-7793.1998.803bg.x 9714861 PMC2231157

[B127] Shmigol A. V. Eisner D. A. Wray S. (2001). Simultaneous measurements of changes in sarcoplasmic reticulum and cytosolic. J. Physiol. 531, 707–713. 10.1111/j.1469-7793.2001.0707h.x 11251052 PMC2278495

[B128] Shmygol A. Gullam J. Blanks A. Thornton S. (2006). Multiple mechanisms involved in oxytocin-induced modulation of myometrial contractility. Acta Pharmacol. Sin. 27, 827–832. 10.1111/j.1745-7254.2006.00393.x 16787565

[B129] Simmons L. V. E. Rubens C. E. Darmstadt G. L. Gravett M. G. (2010). Preventing preterm birth and neonatal mortality: Exploring the epidemiology, causes, and interventions. Semin. Perinatol. 34, 408–415. 10.1053/j.semperi.2010.09.005 21094415

[B130] Smith R. D. Babiychuk E. B. Noble K. Draeger A. Wray S. (2005). Increased cholesterol decreases uterine activity: Functional effects of cholesterol alteration in pregnant rat myometrium. Am. J. Physiol. Cell Physiol. 288, C982–C988. 10.1152/ajpcell.00120.2004 15613497

[B131] Smith R. Imtiaz M. Banney D. Paul J. W. Young R. C. (2015). Why the heart is like an orchestra and the uterus is like a soccer crowd. Am. J. Obstet. Gynecol. 213, 181–185. 10.1016/j.ajog.2015.06.040 26116101

[B132] Szal S. E. Repke J. T. Seely E. W. Graves S. W. Parker C. A. Morgan K. G. (1994). [Ca2+](i) signaling in pregnant human myometrium. Am. J. Physiol. 267, E77–E87. 10.1152/ajpendo.1994.267.1.e77 8048517

[B133] Taggart M. J. Burdyga T. Heaton R. Wray S. (1996). Stimulus-dependent modulation of smooth muscle intracellular calcium and force by altered intracellular pH. Pflugers Arch. 432, 803–811. 10.1007/s004240050202 8772130

[B134] Taggart M. J. Menice C. B. Morgan K. G. Wray S. (1997). Effect of metabolic inhibition on intracellular Ca2+ phosphorylation of myosin regulatory light chain and force in rat smooth muscle. J. Physiol. 499, 485–496. 10.1113/jphysiol.1997.sp021943 9080376 PMC1159321

[B135] Taggart M. J. Wray S. (1993a). Occurrence of intracellular pH transients during spontaneous contractions in rat uterine smooth muscle. J. Physiol. 472, 23–31. 10.1113/jphysiol.1993.sp019933 8145141 PMC1160473

[B136] Taggart M. Wray S. (1993b). Simultaneous measurement of intracellular pH and contraction in uterine smooth muscle. Pflugers Arch. 423, 527–529. 10.1007/BF00374951 8351202

[B137] Testrow C. P. Holden A. V. Shmygol A. Zhang H. (2018). A computational model of excitation and contraction in uterine myocytes from the pregnant rat. Sci. Rep. 8, 9159. 10.1038/s41598-018-27069-x 29904075 PMC6002389

[B138] Thijssen K. M. J. Tissink J. G. L. J. Dieleman J. P. Van der Hout - van der Jagt M. B. Westerhuis M. E. M. H. Oei S. G. (2020). Qualitative assessment of interpretability and observer agreement of three uterine monitoring techniques. Eur. J. Obstet. Gynecol. Reprod. Biol. 255, 142–146. 10.1016/j.ejogrb.2020.10.008 33129016

[B139] Tong W. C. Choi C. Y. Karche S. Holden A. V. Zhang H. Taggart M. J. (2011). A computational model of the ionic currents, ca2+ dynamics and action potentials underlying contraction of isolated uterine smooth muscle. PLoS One 6, e18685. 10.1371/journal.pone.0018685 21559514 PMC3084699

[B140] Tong W. C. Tribe R. M. Smith R. Taggart M. J. (2014). Computational modeling reveals key contributions of KCNQ and hERG currents to the malleability of uterine action potentials underpinning labor. PLoS One 9, e114034. 10.1371/journal.pone.0114034 25474527 PMC4256391

[B141] van Gestel I. Ijland M. M. Hoogland H. J. Evers J. L. H. (2003). Endometrial wave-like activity in the non-pregnant uterus. Hum. Reprod. Update 9, 131–138. 10.1093/humupd/dmg011 12751775

[B142] Verhoeff A. Garfield R. E. Ramondt J. Wallenburg H. C. S. (1985). Electrical and mechanical uterine activity and gap junctions in peripartal sheep. Am. J. Obstet. Gynecol. 153, 447–454. 10.1016/0002-9378(85)90085-7 4050919

[B143] Vlemminx M. W. C. Thijssen K. M. J. Bajlekov G. I. Dieleman J. P. Van Der Hout-Van Der Jagt M. B. Oei S. G. (2017). Electrohysterography for uterine monitoring during term labour compared to external tocodynamometry and intra-uterine pressure catheter. Eur. J. Obstet. Gynecol. Reprod. Biol. 215, 197–205. 10.1016/j.ejogrb.2017.05.027 28649034

[B144] Wang I. Politi A. Z. Tania N. Bai Y. Sanderson M. J. Sneyd J. (2008). A mathematical model of airway and pulmonary arteriole smooth muscle. Biophys. J. 94, 2053–2064. 10.1529/biophysj.107.113977 18065464 PMC2257911

[B145] Wanner O. Cranshaw D. J. Pliška V. (1977). The use of dynamic models to study the role of calcium in the oxytocin-induced contractions of the uterus. Mol. Cell. Endocrinol. 6, 281–292. 10.1016/0303-7207(77)90102-2 190065

[B146] Weiss S. Jaermann T. Schmid P. Staempfli P. Boesiger P. Niederer P. (2006). Three-dimensional fiber architecture of the nonpregnant human uterus determined *ex vivo* using magnetic resonance diffusion tensor imaging. Anat. Rec. A Discov. Mol. Cell. Evol. Biol. 288A, 84–90. 10.1002/ar.a.20274 16345078

[B147] Word R. A. Tang D. C. Kamm K. E. (1994). Activation properties of myosin light chain kinase during contraction/relaxation cycles of tonic and phasic smooth muscles. J. Biol. Chem. 269, 21596–21602. 10.1016/s0021-9258(17)31846-x 8063799

[B148] Wray S. Arrowsmith S. (2021). Uterine excitability and ion channels and their changes with gestation and hormonal environment. Annu. Rev. Physiol. 83, 331–357. 10.1146/annurev-physiol-032420-035509 33158376

[B149] Wray S. Burdyga T. (2010). Sarcoplasmic reticulum function in smooth muscle. Physiol. Rev. 90, 113–178. 10.1152/physrev.00018.2008 20086075

[B150] Wray S. (2007). Insights into the uterus. Exp. Physiol. 92, 621–631. 10.1113/expphysiol.2007.038125 17468199

[B151] Wray S. Jones K. Kupittayanant S. Li Y. Matthew A. Monir-Bishty E. (2003). Calcium signaling and uterine contractility. J. Soc. Gynecol. Investig. 10, 252–264. 10.1016/S1071-5576(03)00089-3 12853086

[B152] Wray S. Prendergast C. (2019). The myometrium: From excitation to contractions and labour. Adv. Exp. Med. Biol. 1124, 233–263. 10.1007/978-981-13-5895-1_10 31183830

[B153] Wray S. (1988a). Regulation of intracellular pH in rat uterine smooth muscle, studied by 31P NMR spectroscopy. Biochim. Biophys. Acta 972, 299–301. 10.1016/0167-4889(88)90205-4 3196763

[B154] Wray S. (1988b). Smooth muscle intracellular pH: Measurement, regulation, and function. Am. J. Physiol. 254, C213–C225. 10.1152/ajpcell.1988.254.2.c213 3279796

[B155] Wray S. (1990). The effects of metabolic inhibition on uterine metabolism and intracellular pH in the rat. J. Physiol. 423, 411–423. 10.1113/jphysiol.1990.sp018030 2388156 PMC1189765

[B156] Xu J. Chen Z. Lou H. Shen G. Pumir A. (2022). Review on EHG signal analysis and its application in preterm diagnosis. Biomed. Signal Process. Control 71, 103231. 10.1016/j.bspc.2021.103231

[B157] Xu J. Menon S. N. Singh R. Garnier N. B. Sinha S. Pumir A. (2015). The role of cellular coupling in the spontaneous generation of electrical activity in uterine tissue. PLoS One 10, e0118443. 10.1371/journal.pone.0118443 25793276 PMC4368634

[B158] Yang J. Clark J. W. Bryan R. M. Robertson C. (2003). The myogenic response in isolated rat cerebrovascular arteries: Smooth muscle cell model. Med. Eng. Phys. 25, 691–709. 10.1016/S1350-4533(03)00100-0 12900184

[B159] Yochum M. Laforêt J. Marque C. (2018). Multi-scale and multi-physics model of the uterine smooth muscle with mechanotransduction. Comput. Biol. Med. 93, 17–30. 10.1016/j.compbiomed.2017.12.001 29253628

[B160] Young R. C. (1997). A computer model of uterine contractions based on action potential propagation and intercellular calcium waves. Obstet. Gynecol. 89, 604–608. 10.1016/S0029-7844(96)00502-9 9083321

[B161] Young R. C. Herndon-Smith L. (1991). Characterization of sodium channels in cultured human uterine smooth muscle cells. Am. J. Obstet. Gynecol. 164, 175–181. 10.1016/0002-9378(91)90650-G 1846061

[B162] Young R. C. Smith L. H. Anderson N. C. (1991). Passive membrane properties and inward calcium current of human uterine smooth muscle cells. Am. J. Obstet. Gynecol. 164, 1132–1139. 10.1016/0002-9378(91)90601-M 2014839

[B163] Young R. C. (2015). Synchronization of regional contractions of human labor; direct effects of region size and tissue excitability. J. Biomech. 48, 1614–1619. 10.1016/j.jbiomech.2015.02.002 25698238

[B164] Zhang M. Tidwell V. la Rosa P. S. Wilson J. D. Eswaran H. Nehorai A. (2016). Modeling magnetomyograms of uterine contractions during pregnancy using a multiscale forward electromagnetic approach. PLoS One 11, e0152421. 10.1371/journal.pone.0152421 27019202 PMC4809542

