# Supplementary material for: Modeling and experimental approaches for elucidating multi-scale uterine smooth muscle electro- and mechano-physiology: A review
Source: Front Physiol. 2022 Oct 7;13:1017649. doi: 10.3389/fphys.2022.1017649 (PMC9585314; doi:10.3389/fphys.2022.1017649)
Supplement: Supplementary file 1 [file Table1.docx]

Supplementary Material

# Cell level mechanisms included in mathematical models of the uterine smooth muscle cell.

**Table S1**. Cell-level ion currents and mechanisms for uSMC models published in literature. This list includes the most relevant or cited models (it is not all inclusive). Additional references identify previous models or publications utilized in the model for each column heading; i.e., Rihana, et al. 2009 utilized the Keizer, 1983 PMCA model. Not every mechanism is included with every model; hence we indicate inclusion or exclusion with annotations: the ✓ symbol represents explicit inclusion (algebraic or ODE), † represents implicit inclusion (as a coefficient) and 🗶 indicates omission.

|  | **Bursztyn et al.**  (Bursztyn et al., 2007) | **Rihana et al.**  (Rihana et al., 2009) | **Tong et al.**  (Tong et al., 2014) | **Atia et al.**  (Atia et al., 2016) | **Testrow et al.**  (Testrow et al., 2018) | **Goldzstejn et al.**  (Goldsztejn and Nehorai, 2020) |
| --- | --- | --- | --- | --- | --- | --- |
| **Species Data** | **Rat** | **Rat** | **Rat** | **Human** | **Rat** | **Rat** |
| **Ca^2+^** |  |  | (Tong, 2011 expanded) |  | (Tong, 2011) | (Tong, 2011) |
| **L-Type** | ✓ | ✓ | ✓ | ✓ | ✓ | ✓ |
| **T-Type** | 🗶 | ✓ | ✓ | ✓ | ✓ | ✓ |
| **PMCA** | 🗶 | † (Keizer, 1983) | ✓ | ✓ | ✓ | ✓ |
| **NCX** | 🗶 | † (Keizer, 1983) | ✓ | ✓ | ✓ | ✓ |
| **SERCA** | 🗶 | 🗶 | 🗶 | † | ✓ | 🗶 |
| **Mitochondria** | 🗶 | † (Keizer, 1983) | 🗶 | 🗶 | 🗶 | 🗶 |
| **IP3R** | 🗶 | 🗶 | 🗶 | 🗶 | 🗶 | 🗶 |
| **RYR** | 🗶 | 🗶 | 🗶 | 🗶 | ✓ | 🗶 |
| **SOCE** | 🗶 | 🗶 | 🗶 | 🗶 | ✓ | 🗶 |
| **K^+^** |  |  |  |  |  |  |
| **K_v_, V_m_-gated** | 🗶 | ✓ | √ type 1,2,A, NQ1-4-5 | √ type 2.x,9.x,6.x,4.x,3.x,7.x | √ type 1,2,A, transient | √ type 1,2,A |
| **hERG** | 🗶 | 🗶 | ✓ | ✓ | 🗶 | 🗶 |
| **Kir** | 🗶 | 🗶 | 🗶 | √ type 7.1 | 🗶 | 🗶 |
| **BK, Ca^2+^-gated** | 🗶 | ✓ | √ type a | √ type alpha-beta1, 3, 4 | √ | √ type a |
| **SK** | 🗶 | 🗶 | √ type b | √ type 2, 3, 4 | 🗶 | 🗶 |
| **SLO2.1** | 🗶 | 🗶 | 🗶 | 🗶 | 🗶 | 🗶 |
| **Leak** | 🗶 | ✓ | ✓ | ✓ | ✓ | ✓ |
| **Cl^-^** |  |  |  |  |  |  |
| **ANO1** | 🗶 | 🗶 | ✓ | ✓ | ✓ | ✓ |
| **Back-ground** | 🗶 | 🗶 | 🗶 | ✓ | 🗶 | 🗶 |
| **Na^+^** |  |  |  |  |  |  |
| **V_m_-gated** | 🗶 | ✓ | ✓ | 🗶 | ✓ | ✓ |
| **Other** |  |  |  |  |  |  |
| **Na/K Exchanger** | 🗶 | 🗶 | ✓ | ✓ | ✓ | 🗶 |
| **NSCC** | 🗶 | 🗶 | ✓ | 🗶 | ✓ | ✓ |
| **h** | 🗶 | 🗶 | ✓ | 🗶 | ✓ | ✓ |
| **Na/K/Cl Symporter** | 🗶 | 🗶 | 🗶 | 🗶 | ✓ | 🗶 |
| **Myosin/Contract** | √ (Hai & Murphy) | 🗶 | 🗶 | 🗶 | √ (Hai & Murphy; Yang, 2003) | √ (Hai & Murphy; Yang, 2003 via Testrow, 2018) |

**
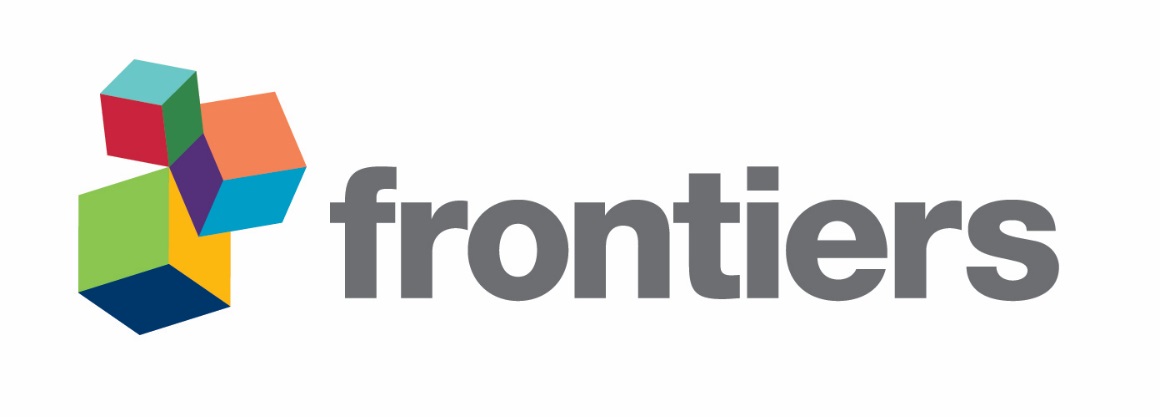
**
